# Human appropriation of net primary production as driver of change in landscape‐scale vertebrate richness

**DOI:** 10.1111/geb.13671

**Published:** 2023-04-10

**Authors:** Karina Reiter, Christoph Plutzar, Dietmar Moser, Philipp Semenchuk, Karl‐Heinz Erb, Franz Essl, Andreas Gattringer, Helmut Haberl, Fridolin Krausmann, Bernd Lenzner, Johannes Wessely, Sarah Matej, Robin Pouteau, Stefan Dullinger

**Affiliations:** ^1^ Department of Botany and Biodiversity Research University of Vienna Vienna Austria; ^2^ Advancing Systems Analysis International Institute for Applied Systems Analysis (IIASA) Laxenburg Austria; ^3^ Institute of Social Ecology (SEC) University of Natural Resources and Life Science (BOKU) Vienna Austria; ^4^ French National Research Institute for Sustainable Development (IRD), AMAP Lab, France & Réunion Marseille France

**Keywords:** biodiversity loss, extinction, human appropriation, land use, net primary production, species–energy relationship, species richness, threatened species

## Abstract

**Aim:**

Land use is the most pervasive driver of biodiversity loss. Predicting its impact on species richness (SR) is often based on indicators of habitat loss. However, the degradation of habitats, especially through land‐use intensification, also affects species. Here, we evaluate whether an integrative metric of land‐use intensity, the human appropriation of net primary production, is correlated with the decline of SR in used landscapes across the globe.

**Location:**

Global.

**Time period:**

Present.

**Major taxa studied:**

Birds, mammals and amphibians.

**Methods:**

Based on species range maps (spatial resolution: 20 km × 20 km) and an area‐of‐habitat approach, we calibrated a “species–energy model” by correlating the SR of three groups of vertebrates with net primary production and biogeographical covariables in “wilderness” areas (i.e., those where available energy is assumed to be still at pristine levels). We used this model to project the difference between pristine SR and the SR corresponding to the energy remaining in used landscapes (i.e., SR loss expected owing to human energy extraction outside wilderness areas). We validated the projected species loss by comparison with the realized and impending loss reconstructed from habitat conversion and documented by national Red Lists.

**Results:**

Species–energy models largely explained landscape‐scale variation of mapped SR in wilderness areas (adjusted *R* ^2^‐values: 0.79–0.93). Model‐based projections of SR loss were lower, on average, than reconstructed and documented ones, but the spatial patterns were correlated significantly, with stronger correlation in mammals (Pearson's *r* = 0.68) than in amphibians (*r* = 0.60) and birds (*r* = 0.57).

**Main conclusions:**

Our results suggest that the human appropriation of net primary production is a useful indicator of heterotrophic species loss in used landscapes, hence we recommend its inclusion in models based on species–area relationships to improve predictions of land‐use‐driven biodiversity loss.

## 1 INTRODUCTION

The survival of biota is increasingly under threat from a rise in the extent and severity of anthropogenic pressures, and land use is currently considered to be the most pervasive driver of biodiversity loss in terrestrial environments (Díaz et al., 2019). Scientific assessments of the impacts that these pressures have on species and ecosystems (i.e., their threat status) are available mostly at global scales. Efforts to classify species threats on national and regional scales are increasing, although the number of countries and/or regions for which such data are available is still limited. The most relevant scale for governance and management of biodiversity, however, is often even finer (e.g., when prioritizing areas for conservation or restoration; Strassburg et al., 2020). Evaluation of biodiversity risks at these finer scales often relies on modelling (e.g., Dullinger et al., 2020; Pereira et al., 2013; Warren et al., 2018), but the representation of land use in these modelling studies still does not fully reflect its importance as a driver of biodiversity loss (Dullinger et al., 2021; Titeux et al., 2016).

Land use can affect biodiversity via many different mechanisms (Dullinger et al., 2021). In addition to data availability issues, this diversity of mechanisms hampers the straightforward inclusion of land use in biodiversity models. When such models are applied at continental to global scales, the most frequently used indicator of land‐use‐related pressure on biodiversity is an area‐based descriptor: habitat loss (Chaudhary & Brooks, 2018; Tilman et al., 2017). This type of modelling is usually based on the species–area relationship, in either its “basic” or modified forms (Pereira et al., 2014). However, using species–area relationships for predictions of species loss faces conceptual issues (He & Hubbell, 2011). Moreover, although some modified versions of species–area models have started to address these shortcomings (e.g., Chaudhary & Brooks, 2018), the focus on habitat loss diverts attention from the fact that land use does not only, and not necessarily, eliminate habitats, but can also degrade habitat quality to a variable extent, with often equally dire effects on biodiversity (Newbold et al., 2015).

There are other well‐known determinants of species richness (SR) that might integrate over both habitat loss and degradation of habitat quality, but have been explored less intensively as possible predictors of human‐induced biodiversity loss. Across terrestrial ecosystems, a major natural gradient that is closely related to SR is biological production, which determines the energy available in ecosystems (Cusens et al., 2012; Wright, 1983). As with the species–area relationship, although some mechanistic explanations have been proposed (Brown, 1981; Wright, 1983), the mechanisms underlying this correlation are not entirely clear (Fine, 2015). Nevertheless, production has been demonstrated to be a strong correlate of spatial biodiversity patterns, in particular at larger (i.e., continental to global) extents (Field et al., 2009; Gaston, 2000). Anthropogenic modification of the level of biological production in ecosystems might thus have a major impact on species (Miko & Storch, 2015; Wright, 1990) and provide an alternative indicator for modelling threats to biodiversity.

An established set of metrics for measuring human‐induced changes of biological production in (terrestrial) ecosystems is provided by the human appropriation of net primary production (HANPP) framework. HANPP is defined as the difference between net primary production (NPP) of unused (potential) natural vegetation under current climatic conditions (NPP_pot_) and the part of NPP that is left in the used ecosystem after harvest (NPP_eco_). HANPP quantifies and integrates changes in the annual carbon flow in ecosystems resulting from both land conversion (which alters biological production of the vegetation, e.g., when converting forest to cropland or infrastructure land) and extraction of plant biomass for human use (through, e.g., crop harvest or livestock grazing; cf. Haberl et al., 2007, 2014; Krausmann et al., 2013; cf. Supporting Information Figure S1.1 in Appendix S1). HANPP metrics have been mapped globally with a landscape‐scale resolution (Haberl et al., 2007; Krausmann et al., 2013) and for long periods of time (Kastner et al., 2022). To assess whether human reduction of biological production is a useful predictor of biodiversity decline at this scale, HANPP metrics should be correlated with the change of SR in these landscapes (i.e., with the difference between current SR and the SR expected in the absence of human activities). The fact that pristine SR of landscapes is rarely known has precluded further exploration of HANPP metrics in this context so far (Haberl et al., 2005).

In this paper, we illustrate a possible way forward in this type of modelling using global patterns of terrestrial vertebrate richness, mapped at a resolution of 20 km × 20 km, as a study system. The approach is based on parameterizing a “species–energy relationship” (SER) in areas assumed to have natural levels of biological production. Subsequently, this relationship is used to project (realized or impending) species loss from landscapes in response to the human appropriation of parts of this production. Given that appropriate fine‐grain data on species loss or threat are not available for the entire globe, we undertake the validation of our projections by reconstructing the already realized and still pending landscape‐scale loss from historical habitat conversion in combination with national Red Lists.

## 2 MATERIALS AND METHODS

Our approach started with parameterizing a species–energy relationship by correlating gridded data (20 km × 20 km, in an equal area projection) of currently realized SR of terrestrial vertebrates to available energy (and biogeographical covariables) in areas assumed to be largely devoid of land use (“wilderness area”). Given that there is neither land conversion nor human harvest in these areas, the NPP_pot_ and NPP_eco_ metrics of available energy are equal there by definition. We then used NPP_pot_ and the fitted relationship to project an estimate of pristine SR (SR_pot_) in non‐wilderness areas, that is in areas where land use currently takes place and the pristine SR has probably changed already in response to these human activities. By substituting NPP_pot_ with NPP_eco_, we subsequently projected the SR expected under the NPP available to wildlife under current land‐use conditions in these non‐wilderness areas. We call this projected SR of non‐wilderness cells SR_lu_ (SR altered owing to land use) and emphasize that it includes impending loss (or gain) of species that might not yet be realized (e.g., as a consequence of an extinction debt or immigration credit). The difference between projected SR_pot_ and SR_lu_ (henceforth ∆SR) thus corresponds to the (already realized or impending) change of SR expected as a result of the change in available energy caused by human appropriation of part of NPP_pot_. Finally, for validation of these projections, we compared ∆SR with the cell‐wise loss of species that we reconstructed from documented site‐specific habitat conversion and habitat affiliations of species in combination with maps of (regionally) extinct species provided by the International Union for the Conservation of Nature (IUCN, 2020) and with national Red Lists of threatened species.

### 2.1 Species richness maps

The richness of a total of 5557 mammal, 11,125 bird and 6666 amphibian species per 20 km × 20 km cell of the global terrestrial surface was determined using an area of habitat approach. The data used were provided by the IUCN (2020) and BirdLife International and Handbook of the Birds of the World (2018). Given that reptile species were represented incompletely in the IUCN Red List, with c. 30% of the species being unassessed at the time we started this analysis, this taxon was not included.

The information on species geographical ranges, habitat affiliations and elevational ranges were combined with maps on potential pristine ecosystems, remotely sensed (current) land cover data, and a digital elevation model (Brooks et al., 2019). This approach provides a more accurate estimate of a species distribution than simply relying on unfiltered extent‐of‐occurrence range maps, because gaps attributable to a lack of suitable habitat or elevation are accounted for (Brooks et al., 2019; Strassburg et al., 2020).

To assemble SER models (see section 2.4) and to compare model projections to reconstructed realized and impending loss, we calculated three distinct SR maps, separately for each taxonomic group (Table 1). The first SR map included all observed, currently extant species per cell (SR_obs_), the second included all species assumed to have gone extinct at a site (SR_ext_), and the third included the subset of those species from SR_obs_ currently categorized as threatened in areas covered by national or regional Red Lists (SR_threat_). We describe the computation of these maps in detail in the Supporting Information (Supplementary Methods in Appendix S1).

**TABLE 1:** Description of different species richness maps derived from data sources or projected from models.

Variable name	Description
SR_obs_	Observed species richness [i.e., all species listed as (1) extant or probably extant, (2) native or reintroduced, and (3) resident or present during the breeding season or the non‐breeding season and filtered for matching ESA‐CCI Land Cover category, Olson Biome and elevation range]
SR_threat_	Number of threatened species (i.e., subset of SR_obs_ categorized as threatened in national/regional Red Lists)
SR_ext_	Number of extinct species [i.e., all species listed as extinct (post 1500) or possibly extinct in IUCN and BirdLife databases + the difference in species numbers when filtering range maps only by the pristine habitat vs. calculated with filtering by both the pristine and the current habitats of a site]
SR_pot_	In wilderness areas: SR_pot_ = SR_obs_ + SR_ext_ In non‐wilderness areas: Modelled species richness in absence of human appropriation of net primary production
SR_lu_	Modelled species richness left after the modification of the level of net primary production by humans
∆SR	Modelled species richness loss (i.e., SR_pot_ − SR_lu_)

### 2.2 HANPP metrics

The approach taken here requires a comparison of two different NPP indicators, the difference of which is defined as HANPP (Haberl et al., 2014): (1) the NPP that would prevail in a hypothetical case without land use [i.e., the NPP of a (hypothetical) pristine vegetation, but with current climate (NPP_pot_)]; and (2) the NPP available to organisms after humans have converted this vegetation to cultivated land and extracted NPP through harvest or livestock grazing (NPP_eco_):

$${\mathrm{NPP}}_{\mathrm{eco}}={\mathrm{NPP}}_{\mathrm{pot}}–\text{HANPP}.$$

NPP_pot_ can be modelled using dynamic global vegetation models. In our case, maps on NPP_pot_ were calculated with LPJ‐GUESS (Smith et al., 2014) v.4.0.1., using its standard configuration but without nitrogen limitation and CRU‐NCEP climate forcing data (Harris et al., 2014). NPP_eco_ was calculated from estimates of the NPP level of the vegetation after land‐use change has taken place (HANPP_luc_) and the amount of harvested NPP (HANPP_harv_; Supporting Information Figure S1.1 in Appendix S1; Haberl et al., 2014). In other words, the difference between NPP_pot_ and NPP_eco_ (i.e. HANPP) is the sum of HANPP_harv_ and HANPP_luc_:

$$\text{HANPP}={\text{HANPP}}_{\text{harv}}+{\text{HANPP}}_{\mathrm{luc}}.$$

We derived NPP_eco_ by subtracting HANPP from NPP_pot_, largely following the routines described by Haberl et al. (2007), Krausmann et al. (2013) and Semenchuk et al. (2022). For a description of datasets and methods, see Supporting Information (Supplementary Methods; for maps on the spatial pattern of NPP_pot_, NPP_eco_ and HANPP, see Supporting Information Figure S1.2 in Appendix S1). All these metrics were calculated and mapped for the year 2011 (as an average of the period between 2009 and 2013) and aggregated from a resolution of 5′ to a resolution of 20 km × 20 km. Grid cells in unproductive areas (Antarctica, Greenland or deserts, such as parts of the Sahara) were treated as no‐data zones owing to their very low natural productivity levels.

### 2.3 Biogeographical variables

We controlled for historical, biogeographical legacies on SR patterns by including zoogeographical realms, as mapped by Holt et al. (2013), as a covariate in the SER models (see section 2.4; cf. Supporting Information Figure S1.2 in Appendix S1). One of these zoogeographical realms (Madagascar) contains no wilderness areas, which made inferences about SR loss there impossible. Therefore, we treated the Madagascan realm as a no‐data zone and excluded it from our calculations. Additionally, we differentiated between continents and islands to account for isolation‐dependent differences in SR. Given that preliminary model predictions resulted in overestimations of SR for the Greater Sunda Islands, this archipelago, which was connected over extended time periods with Southeast Asia during the ice ages, was considered continental in the final SER models.

All environmental datasets were converted to an Eckert IV projection at a resolution of 20 km × 20 km. Variables available at a higher resolution were aggregated to 20 km × 20 km either by majority resampling, in the case of factorial variables such as zoogeographical realms or habitat types, or bilinear interpolation, in the case of continuous variables.

### 2.4 Statistical analyses

To calibrate SER models that are not affected by human activity, we constrained model fitting to wilderness areas [i.e., 20 km × 20 km landscapes with (almost) no land use]. We distinguished between wilderness and non‐wilderness areas using a combination of datasets: the human footprint, (i.e., a global map of the density of human artefacts for the years 1993 and 2009; Venter et al., 2016a, 2016b) and global maps depicting areas of intact forest landscapes for the years 2000 and 2013 (Potapov et al., 2008; Potapov et al., 2017). Outside of forests, areas with zero human footprint were categorized as wilderness areas. Potentially forested areas were classified as wilderness only if they showed zero human impact and were part of an intact forest.

To relate SR to NPP_pot_, the biogeographical region (a factor variable with 10 levels) and mainland–island identity (a factor with two levels), we drew a stratified sample from the wilderness areas. We selected 500 cells of 20 km × 20 km from each of the considered 10 zoogeographical realms. Given that wilderness areas are not distributed evenly across the terrestrial ecosystems of the Earth, three realms had <500 wilderness cells (for maps on the spatial distribution of wilderness areas across the globe and the terrestrial zoogeographical realms, see Supporting Information Figure S1.2 in Appendix S1). In these cases, one‐third of the wilderness cells of the respective realms were selected. We restricted selection to one‐third of all cells to reduce the effect of spatial autocorrelation as far as possible. To avoid extrapolation of the relationships beyond the sampled predictor range, the sample was forced to include the cells with minimum and maximum values of NPP_pot_ (across all zoogeographical realms). The final sample created in this way comprised 3575 cells.

For each of these 3575 cells, we first summed the number of extant (SR_obs_) and extinct (SR_ext_) species to account for possible extinctions of species in these areas for reasons other than land use (e.g., hunting), resulting in the potential SR (SR_pot_). We then related SR_pot_ as the response variable to our predictor variables by means of multiple quasi‐Poisson regression (to account for possible overdispersion in the count data) separately for each of the three taxonomic groups. We assumed a linear effect of NPP_pot_ on the response that is the same across all biogeographical realms, but allowed for different intercepts per realm or on mainlands and islands. The predictor of the regression was thus an additive combination of NPP_pot_, biogeographical realm and mainland–island identity. The variation uniquely explained by NPP_pot_ and other variables was assessed by calculating partial *R* ^2^‐values using the R package *rsq* (v.2.2; Zhang, 2021). We evaluated the uncertainty of projections from the model by projecting expected species numbers in pristine conditions (SR_pot_) across all terrestrial surfaces, thereby varying the regression coefficient of NPP_pot_ within the interval defined by its standard error.

The fitted SR_pot_ models were then used to compute the number of species expected to be lost per cell in response to human reduction in available NPP outside wilderness regions. To do so, we first projected expected species numbers in pristine conditions (SR_pot_; i.e., under assumed absence of land use) across all 288,770 cells, using NPP_pot_ (and all other covariates) as predictors. Second, we repeated the same projections, replacing NPP_pot_ with NPP_eco_ to calculate the expected number of species with the energy available under current land use (SR_lu_). The differences in the projected species numbers per site, SR_pot_ − SR_lu_ (∆SR), were based entirely on the partial effect of NPP in the SER models. To assess whether projections of SR_lu_ fitted the distribution of SR calculated from species range maps and habitat affiliations (SR_obs_), we compared SR_lu_ and SR_obs_ by means of Pearson correlation coefficients. Correlations were computed for 10 random samples of cells from non‐wilderness areas (*n* = 5000 per draw). We additionally calculated linear regression models of SR_lu_ versus SR_obs_. We then used the deviation of the estimated slope of this relationship from one to evaluate whether projections of SR_lu_ under‐ or overestimated values of SR_obs_.

We considered the difference between SR_pot_ and SR_lu_ (∆SR) as the metric representing expected (realized or pending) species loss resulting from the human‐induced reduction of NPP available in ecosystems. To assess whether patterns of SR loss calculated in this way matched patterns of reconstructed realized and impending species loss, we calculated Pearson correlation coefficients between the added maps of SR_ext_ and SR_threat_ (SR_thr+ext_) and ∆SR. Correlations were computed for 10 random samples of cells (*n* = 5000 per draw) drawn from those areas where national/regional Red Lists were available. Given that we focused on SR loss trigged by human land use, we drew these samples from non‐wilderness areas only (because predicted loss is zero in wilderness areas by definition, given that NPP_pot_ = NPP_eco_). We also calculated linear regression models of ∆SR vs. the reconstructed species loss. We then used the deviation of the estimated slope of this relationship from one to evaluate whether projections of ∆SR under‐ or overestimated reconstructed species loss.

All statistical analyses were performed using R v.4.1.1 (2021‐08‐10; R Core Team, 2021).

## 3 RESULTS

### 3.1 Species–energy models and projections of actual species richness

Our SER model fitted in wilderness areas explained the distribution of SR in these wilderness areas very well. The models accounted for 89% of the variance of species numbers for birds, 93% for mammals and 79% for amphibians (cf. Supporting Information Figures S.14 and S.15 in Appendix S1). All predictor variables had significant effects on SRexcept for the mainland–island variable in amphibians and a particular biogeographical realm in birds (realm 7; Supporting Information Tables S.1.1–S1.3 in Appendix S1). Based on *t*‐values, available energy (NPP_pot_) had the strongest effect, affecting predictions of the model more strongly than any individual biogeographical covariable. However, the high partial *R* ^2^‐values of the covariate “zoogeographical realm” as a whole in the models of all three taxonomic groups indicated that biogeographical history is a very important co‐determinant of SR. Projected pristine species richness (SR_pot_) outside wilderness areas was robust against using different values of the estimated regression coefficient of NPP_pot_ within the range spanned by its standard error (Supporting Information Figure S1.4 in Appendix S1). Although predicted maximum species numbers varied slightly (mammals and amphibians) to moderately (birds), geographical patterns of SR_pot_ remained remarkably stable.

**FIGURE 1:**
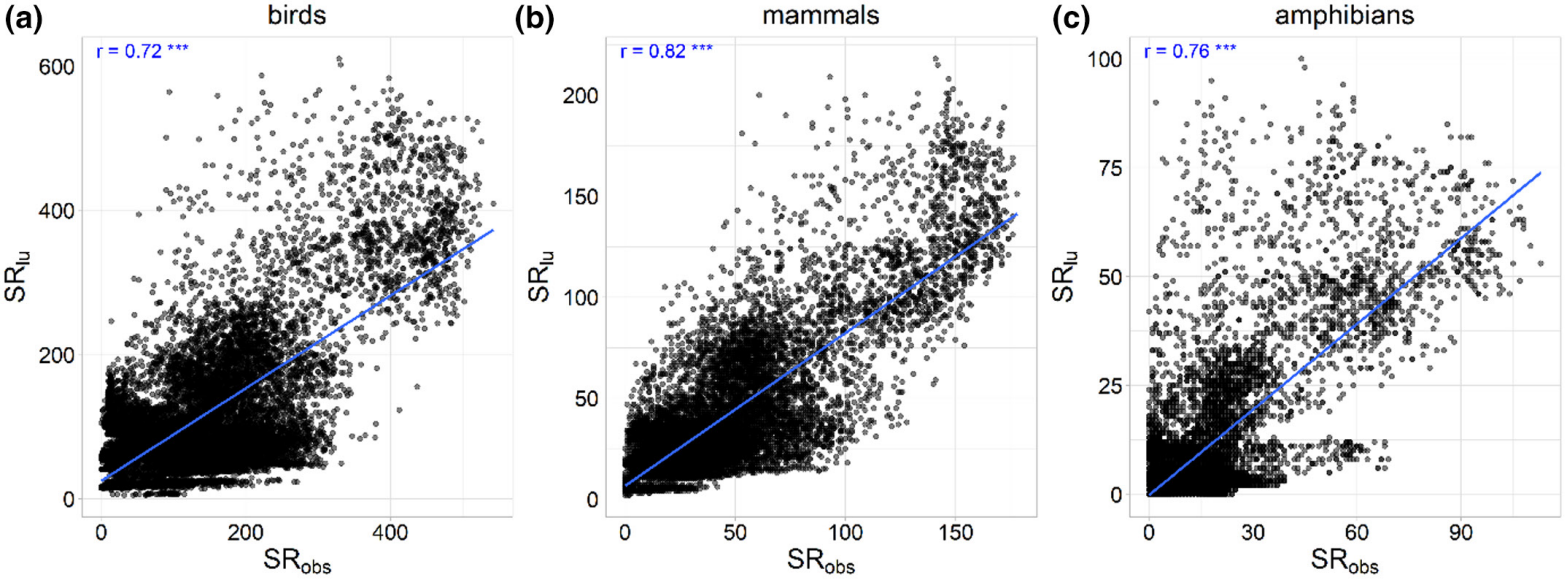
The relationship between observed (SR_obs_) and predicted actual species richness (SR_lu_) across 20,000 randomly selected cells of 20 km × 20 km for (a) birds, (b) mammals and (c) amphibians. The blue line represents a regression line, and the annotations give the correlation coefficients (Pearson's *r*) and their *p*‐values (***p < 0.001).

**FIGURE 2:**
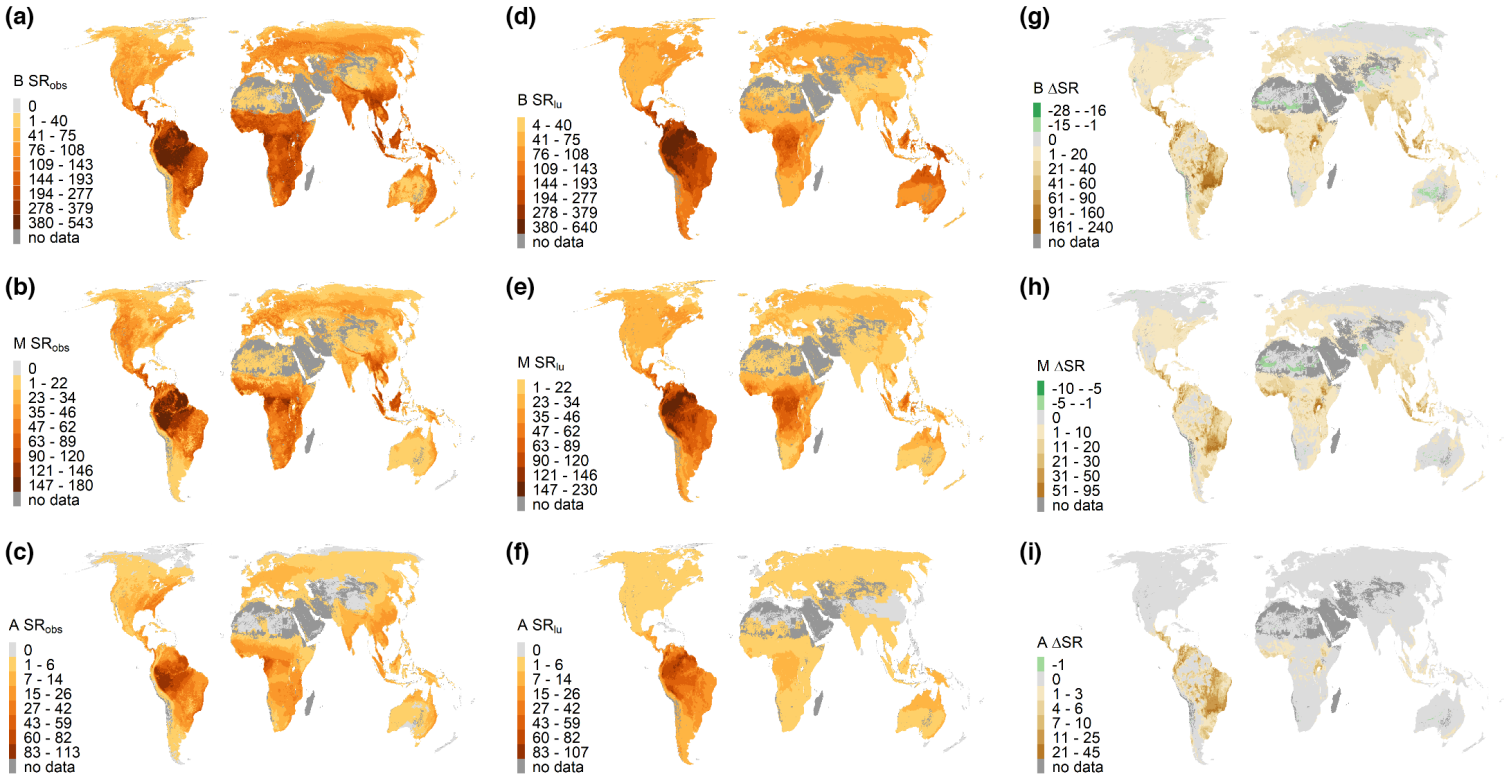
(a–f) Maps of the number of observed species richness (SR_obs_) and the actual species richness as projected by SER models (SR_lu_) for (a,d) birds (B), (b,e) mammals (M) and (c,f) amphibians (A). (g–i) Maps show the difference between SR_pot_ (= SR under NPP_pot_; Supporting Information Figure S1.3 in Appendix S1) and SR_lu_ (i.e., ∆SR) for (g) birds, (h) mammals and (i) amphibians. Negative ∆SR values indicate a predicted gain in SR, whereas positive ∆SR values show losses in SR.

Outside wilderness areas, the relationship between projected SR_lu_ and the number of extant species calculated from range maps and habitat affiliations (SR_obs_) was also strong (*r* = 0.72 in birds, *r* = 0.82 in mammals and *r* = 0.76 in amphibians, *p* < .001 for each taxonomic group at a sample size of 20,000), although with considerable scatter (Figures 1 and 2a–c). Across taxonomic groups, this scatter had some consistent geographical pattern. In particular, SR_lu_ was lower than SR_obs_ across all taxonomic groups in sub‐Saharan Africa, Southeast Asia, China, India and Nepal, especially along the southern slopes of the Himalayas (Figure 2). The slope of the linear regression between SR_lu_ and SR_obs_ had values lower than one in all groups (birds = 0.60, mammals = 0.82 and amphibians = 0.81), indicating that the projections tended to underestimate SR_obs_, especially in the case of birds (Figure 1).

### 3.2 Projections of species loss

Geographical patterns of ∆SR (i.e., the difference between projected pristine species SR_pot_ and projected actual/current species richness SR_lu_ outside wilderness areas) were similar across taxonomic groups, especially between mammals and birds (Figure 2g–i). Hotspots of projected species loss clustered in large parts of Central and South America, Southeast Asia and India, areas around the African Great Lakes and along the West African coast, and southern Florida. Gains in SR (i.e., negative ∆SR values) were recorded for areas where NPP_eco_ values surpass those of NPP_pot_, mostly as a result of fertilization and irrigation in arid regions, such as parts of Sahara, the Nile Delta, the Indus valley, some coastal regions of Peru or parts of Australia.

**FIGURE 3:**
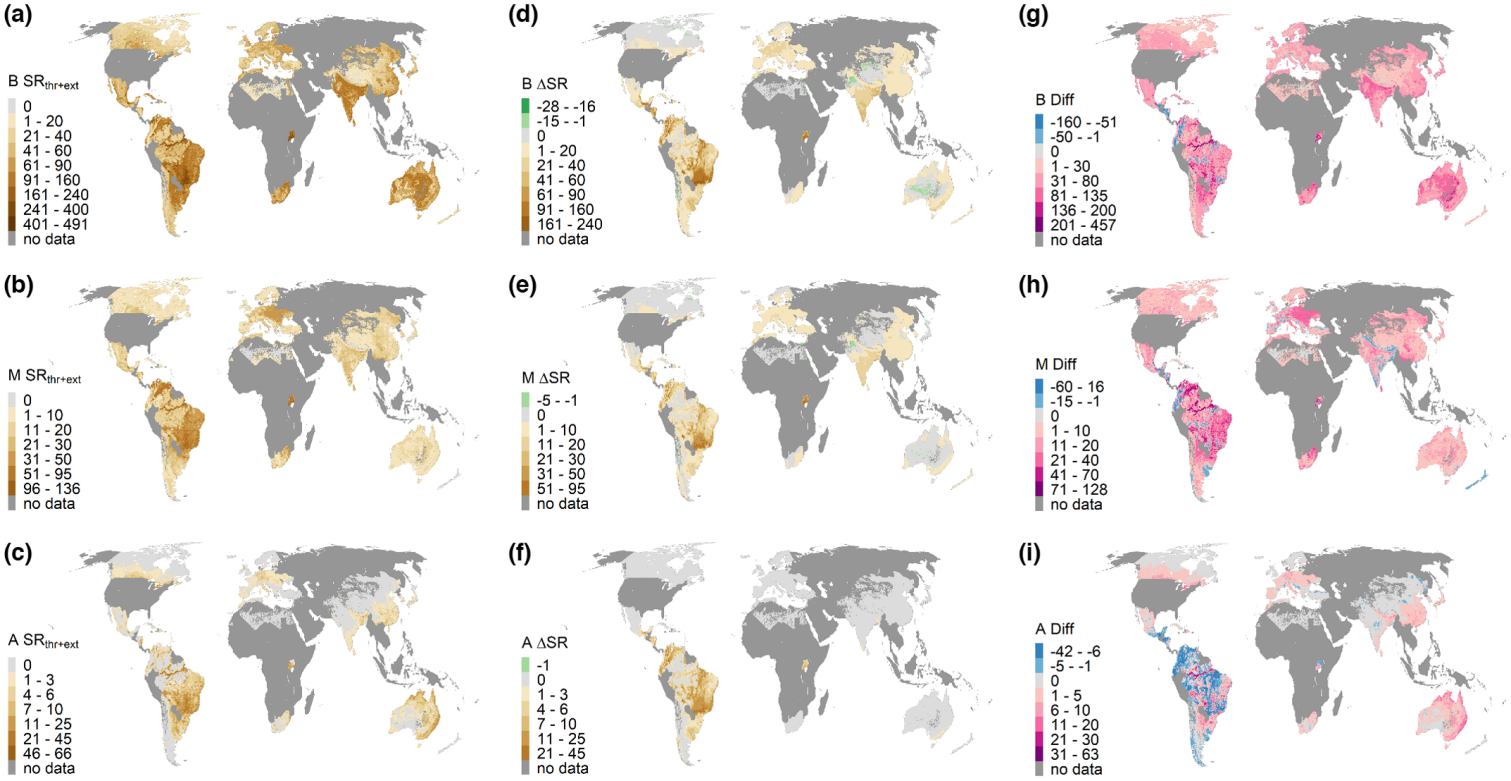
Spatial patterns of the realized and impending species loss reconstructed from the combination of habitat conversion, national Red Lists and IUCN maps of extinct species (SR_thr+ext_) in those areas where national/regional Red Lists were available for (a) birds (B), (b) mammals (M) and (c) amphibians (A). (d–f) ∆SR [i.e., the difference between SR_pot_ (Supporting Information Figure S1.3 in Appendix S1) and SR_lu_] for these same areas. (g–i) Difference between SR_thr+ext_ and ∆SR (i.e., SR_thr+ext_ − ∆SR). Blue areas indicate over‐prediction of modelled ∆SR compared with SR losses observed by the IUCN (i.e., areas in which ∆SR > SR_thr+ext_), whereas pink areas depicting positive values are those in which our SER model underestimates SR losses (i.e., ∆SR < SR_thr+ext_). Dark grey areas are those for which national or regional Red Lists are not available, in addition to non‐productive areas.

**FIGURE 4:**
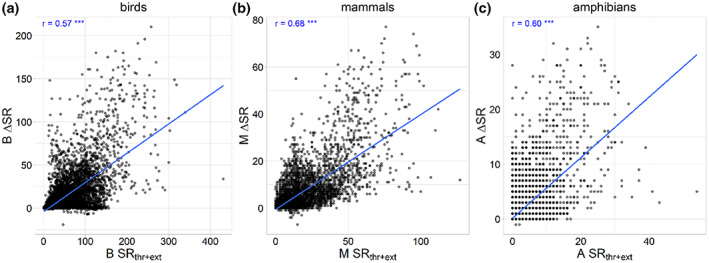
The relationship between projected SR change (∆SR) and the realized and impending species loss reconstructed from the combination of habitat conversion, national Red Lists and IUCN maps of extinct species (SR_thr+ext_) in 5000 cells of 20 km × 20 km randomly selected in non‐wilderness areas for (a) birds (B), (b) mammals (M) and (c) amphibians (A). The blue line represents a regression line, and the annotations give the correlation coefficients (Pearson's *r*) and their *p*‐values (***p < 0.001).

Numbers of modelled ∆SR values were lower than the number of reconstructed realized and impending species loss (SR_thr+ext_) across most of the globe (Figure 3). Only for amphibians ∆SR exceeded SR_thr+ext_ across larger areas, especially in South America. The spatial patterns of these two metrics in non‐wilderness areas (and within those areas for which national or regional Red Lists are available) were significantly correlated (*p* < 0.001 throughout) in all three taxonomic groups. Relationships were closest in mammals (*r* = 0.68) and somewhat less pronounced in amphibians (Person's *r* = 0.60) and birds (*r* = 0.57). Linear regression models of ∆SR and SR_thr+ext_ had slopes considerably below one for all groups (birds = 0.33, mammals = 0.40 and amphibians = 0.55; Figure 4), corroborating the predominant under‐prediction of ∆SR in comparison to SR_thr+ext_.

## 4 DISCUSSION

Net primary production, in combination with biogeographical covariables, explains a large percentage of the landscape‐scale variation of vertebrate SR in wilderness areas. These results are in line with a long tradition of research on the species–energy relationship (e.g., Hawkins et al., 2003; Luck, 2007). The extent to which this relationship is correlational or mechanistic is contentious (Fine, 2015). At least for heterotrophic organisms, however, a mechanistic component is plausible, because reduction of available energy affects population sizes, hence survival probability of local or regional populations (Brown, 1981; Evans et al., 2005; Hanski, 2011; Miko & Storch, 2015; Wright, 1983).

Problems of mechanistic interpretation also propagate to predictions of species loss in response to the human energy extraction. Patterns of HANPP covary with other aspects linked to human land use and its intensity (Erb et al., 2013), such as habitat loss and fragmentation (Seibold et al., 2019; Wearn et al., 2012), human disturbance (Blüthgen et al., 2012) or pollution by agrochemicals (Firbank et al., 2003). In part, this covariation is captured implicitly by the metrics of the HANPP framework, because the difference between NPP_pot_ and NPP_eco_ measures energy loss as a result of both extracted biomass and changed production of the converted ecosystem. However, the covariation of several potential drivers of biodiversity loss hinders the disentangling of their relative impacts and attributing species loss, or a certain fraction of it, to the loss of energy per se. On the contrary, this integrative aspect makes HANPP potentially more widely applicable as an indicator of land‐use‐driven species decline than approaches based on habitat loss alone. This is particularly valid wherever land use degrades, but does not convert natural ecosystems (i.e., where usage reduces habitat quality without altering the type of the ecosystem, such as for natural forests used for timber production or natural grasslands used for livestock grazing; Chaudhary et al., 2016; Erb et al., 2018). Such used, but unconverted ecosystems are widespread across the globe (Arneth et al., 2019; Erb et al., 2017; Semenchuk et al., 2022). This advantage of HANPP as an integrative measure of land‐use‐driven species loss might trade off against the fact that consequences of habitat loss for biodiversity, which are strong in many contexts (Pereira et al., 2014), are only implicitly reflected in HANPP metrics and that there might be situations where NPP levels remain similar despite ecosystem conversion. Further development of biodiversity models should therefore focus on combining aspects of habitat loss with indicators of habitat degradation; for instance, by merging approaches that are based on the reduction of available area with those focusing on the reduction of available energy (Semenchuk et al., 2022).

Despite uncertainties of mechanistic interpretation, projections of species loss based on our species–energy model are correlated with the realized and impending species loss reconstructed from independent data sources. The HANPP approach thus provides us with useful indicators of land‐use effects on biodiversity. Of course, uncertainties relate to the construction of HANPP maps. Although the HANPP framework is built in a way that uses state‐of‐the‐art datasets, a thorough ground‐truthing remains impossible. However, the HANPP approach integrates all datasets in a meaningful and consistent framework, in order to close data gaps and minimize the effect of these uncertainties (Haberl et al., 2007; Krausmann et al., 2008, 2013). Data inter‐comparisons have shown that overall estimates and patterns of these types of HANPP assessments are relatively robust (Kastner et al., 2022). Our findings thus support the usage of HANPP measures in the spatial planning of biodiversity management. Spatially explicit information can, for example, contribute to the development of prioritization schemes for area‐based conservation (e.g., Jung et al., 2021) or ecosystem restoration (Strassburg et al., 2020) by indicating the degree to which pristine diversity has probably already been depleted by human land use. A practical advantage of HANPP in this respect is that this metric is readily available at global and continental scales and comparatively fine spatial resolutions (e.g., Kastner et al., 2022; Plutzar et al., 2016). Moreover, HANPP_harv_ is directly related to one central purpose of land use(i.e., the provision of biomass as an indispensable resource for social metabolism), hence use of this approach contributes to a better understanding of the coupled socio‐ecological system (Haberl et al., 2019).

Nevertheless, the correlations we found between ∆SR and SR_thr+ext_ were far from perfect. A number of different factors are likely to have contributed to this lack of fit. First, land use is only one among several human activities that drive species threat (Díaz et al., 2019). Confounding effects from drivers other than land use are particularly likely in the case of amphibians, where many species are more directly at risk from the spread of *Batrachochytrium dendrobatidis*. This chytrid fungus has also encroached into relatively pristine areas and threatens species there, independent of human land‐use patterns (Hof et al., 2011; Scheele et al., 2019). In mammals and birds, direct persecution is known to be an important threat in various regions of the world, including areas that are yet little affected by land use (Tilman et al., 2017). Second, HANPP does not capture all the different processes by which land use can affect species populations, as already discussed above (Dullinger et al., 2021). Third, there are many data issues affecting the accuracy of both projected ∆SR and SR_thr+ext_. To mention only a few, there are challenges in distinguishing unused (= wilderness) and used areas with remote sensing (Erb et al., 2007; Riggio et al., 2020). Moreover, there are clear sampling biases: most wilderness areas are found in high latitudes or in (sub)tropical areas (e.g., inland Australia and Amazon forest), whereas temperate regions with high human population density are underrepresented. As a consequence, data support for the relationship between NPP (and the other variables in our SER models) and SR is weaker for those parts of the globe that have undergone the most severe transformation of their ecosystems (see Supporting Information Figure S1.5 in Appendix S1). Fortunately, remaining wilderness areas cover the extremes of NPP_pot_ well, hence effects of those data biases on the species–energy models are probably rather moderate. Furthermore, documentation of species distribution and species threat is also heterogeneous across geographical regions (Titley et al., 2017). As a consequence, national and regional Red List data are not available for large parts of the Earth (see Figure 3). It is unclear whether the relationship between ∆SR and SR_thr+ext_ would differ from what we found if the regions currently uncovered by national Red Lists could be included. Finally, the strength of the correlations found might be attributable, in part, to an underlying common relationship between the SR of an area and ∆SR in addition to SR_thr+ext_. However, given that conservation has a primary interest in the number of species lost or threatened, a model that can predict these numbers well is of high value, even if parts of its accuracy are attributable to underlying patterns of pristine SR.

Apart from the scatter in the relationship between ∆SR and SR_thr+ext_, there also appears to be a systematic bias, with ∆SR being considerably lower, on average, than SR_thr+ext_. Given that both of these measures of species loss are estimates based on models and/or expert opinion, it is difficult to distinguish which of them under‐ or overestimates the “real” biodiversity effects of land use. On the one hand, not all national Red Lists comprise a comprehensive representation of all species of a taxonomic group in the country. Systematic information on the comprehensiveness of these lists is hard to obtain, but lack of completeness will result in underestimation of SR_thr+ext_ in the respective areas. On the other hand, there are also a number of factors that will have boosted SR_thr+ext_. First, Red Lists deliver information on whether species are threatened over a spatial domain larger than the one we target with our models, and threat at the national level does not necessarily imply threat at the landscape level. Second, our reconstruction of SR_thr+ext_ accounts only for the loss of species from the pristine species pool of a cell, but not for possible colonization of species new to the cell (i.e., not affiliated with the pristine habitat) after habitat conversion, for example by grassland species after deforestation. Where such colonization has occurred, SR_thr+ext_ will overestimate the realized decrease in species numbers. Third, although the inclusion of threatened species is sensible to account for extinction debts, not all threatened species will necessarily go extinct over time even if conditions remain as they are. Fourth, we have simplified real habitat mosaics by assigning each 20 km × 20 km cell only one habitat (i.e., the dominant habitat type). This simplification will have resulted in overestimation of SR_thr+ext_ where the pristine habitat has been reduced in a cell, but where remnants of it are still available. Concerning modelled species loss, ∆SR, it might underestimate real species loss if the underlying species–energy model underestimates the sensitivity of SR to the amount of available energy (i.e., the regression coefficient of NPP_pot_; cf. Supporting Information Figure S1.5 in Appendix S1) or where humans change the habitat without reducing available energy, as seen most clearly in irrigated arid lands. It will, in contrast, overestimate species loss if humans extract large amounts of energy without converting the habitats, as in intensively used primary forest or grasslands. Taken together, we speculate that part of the systematic difference between ∆SR and SR_thr+ext_ is attributable to a net overestimation of SR_thr+ext_, although the magnitude of this methodological bias cannot be quantified with the data at hand.

## 5 CONCLUSION AND RECOMMENDATIONS

Land use currently affects approximately three‐quarters of the ice‐free land mass of the Earth (Arneth et al., 2019) and will probably increase further in extent and, in particular, in intensity as a response to a rise in human population, economic growth and changes in lifestyle and diets (Ellis et al., 2013; Foley et al., 2011). Estimating the impending species losses not only at the global scale, but also at landscape scales is thus increasingly important, because it is at this scale where biodiversity loss translates into loss of ecosystem services (Cardinale et al., 2012) and where area‐based conservation measures are most often implemented (Maxwell et al., 2020). Models for predicting species losses have so far often been based on species–area relationships. Here, we show that indicators of human‐induced changes of NPP are significantly correlated with species loss in used landscapes and thus offer a valuable alternative approach to establish such models. HANPP indicators directly capture the impact of changes in the availability of trophic energy on heterotrophic food chains and are a compound correlate of many ecologically relevant processes associated with land use. A particular advantage of HANPP indicators is that they capture changes in land‐use intensity, in particular the degradation of landscapes not explicitly converted to agroecosystems, which cannot be considered in models that rely exclusively on measures of habitat loss. However, we do not suggest that NPP‐based models should replace those based on habitat loss. Rather, we advocate an expansion of the modelling toolbox to exploit the complementarities of different approaches, ideally towards an integration of several indicators which, in combination, could cover the various processes through which land use affects biodiversity.

## CONFLICT OF INTEREST

We have no conflicts of interest to disclose.

## BIOSKETCH

The author team consists of researchers with diverse backgrounds, including ecology, sustainability science and environmental research, whose interests lie in the collaboration of natural and social sciences to gain a better understanding of the effects of global changes and socio‐economic activities on natural systems across space and time. With the knowledge gained, this team wants to advance current environmental and sustainability research and provide a well‐founded basis for decision‐making for conservation management and sustainable development.

## Supporting information


**Appendix S1.** Supporting information.

## Data Availability

Geographical range data for terrestrial vertebrates were obtained from the IUCN Red List of Threatened Species, Version 2020‐2, openly available at https://www.iucnredlist.org/resources/grid/spatial‐data and BirdLife International and Handbook of the Birds of the World (2018). Datasets on the human footprint were obtained from the DRYAD repository openly available at https://datadryad.org/stash/dataset/doi:10.5061/dryad.052q5. Datasets on intact forest landscapes were obtained from the global IFL repository openly available at http://www.intactforests.org. Datasets on zoogeographic realms were obtained from the CMEC Zoogeographic Realms and Regions repository openly available at https://macroecology.ku.dk/resources/wallace. Datasets on global climate were obtained from the CHELSA climate data repository openly available at https://chelsa‐climate.org/downloads/. The map denoting mainland‐island identity is available within the article's supplementary materials. Datasets on global HANPP are available within the article's supplementary materials. For questions concerning this data please contact sarah.matej@boku.ac.at or thomas.kastner@senckenberg.de.
