# Supplementary material for: Human appropriation of net primary production as driver of change in landscape‐scale vertebrate richness
Source: Glob Ecol Biogeogr. 2023 Apr 10;32(6):855–66. doi: 10.1111/geb.13671 (PMC10946509; doi:10.1111/geb.13671)
Supplement: Supplementary file 1 — Appendix S1. Supporting information. [file GEB-32-855-s001.zip › GEB_13671_Supplementary_Information.docx]

# Supplementary Information

## Supplementary Methods

#### **1.1 Computing species richness maps**

To assemble SER models (see below) and to compare model projections to reconstructed realized and impending loss, we separately calculated three distinct SR maps for each taxonomic group. The first SR map includes all observed, currently extant species per cell (SR_obs_), and is based on each species’ range polygons provided by the IUCN (2020) and BirdLife International and Handbook of the Birds of the World (2018). We only included part of the range where the species is classified as (i) extant or probably extant, (ii) native or reintroduced, and (iii) resident or present during the breeding season or the non-breeding season. The maps were rasterized using a 20 x 20 km reference grid and the *terra* package in R (Hijmans 2021). We then used all the species’ gridded range maps to create a species list for each 20 x 20 km cell using the *velox* package in R (Hunziker 2018). From these cell-wise lists we excluded: (i) species exclusively affiliated with aquatic habitats; (ii) species unaffiliated with the (potential) pristine habitat associated with each cell. To do so, we assigned the terrestrial habitats listed in the species’ habitat affiliation schemes of the IUCN (2020) and BirdLife International and Handbook of the Birds of the World (2018) to one or several of the 14 pristine ecosystem types distinguished in the global maps in Dinerstein et al. (2017) (see SI Table S1.4; smallest polygon distinguished by Dinerstein et al. (2017): 3 km² ); (iii) species unaffiliated with the current habitat of the cell as defined via habitat maps provided by the ESA-CCI land cover time series v2.0.7 (using data from the year 2011) from the Climate Research Data Package (CRDP, ESA 2017, spatial resolution: 300m, upscaled to 20 x 20 km by a majority filter; see SI Table S1.5 for the cross-classification scheme linking species’ habitat affiliation to the habitat types distinguished in ESA-CCI). The combination of the latter two filtering steps implies that possible colonization of a cell by species affiliated with the current conditions but not with the pristine ones was not accounted for. We did so, because loss of species can be considered a necessary consequence of habitat loss, while colonization of newly available habitat might or might not occur. We emphasize, however, that this approach will tend to underestimate real species richness in at least part of the cells; (iv) species whose elevational range (taken from the IUCN (2020) and BirdLife International and Handbook of the Birds of the World (2018)) does not overlap with the elevational range of the grid cell as calculated from the GMTED2010 dataset (www.usgs.gov).

The second SR map includes all species assumed to have gone extinct at a site (SR_ext_). The list of extinct species was the sum of two components. First, we used the range polygons of the IUCN and Birdlife databases delineating the areas where the species is classified as extinct (post 1500) or possibly extinct (given that it was (i) native or reintroduced or (ii) resident or present during the breeding season or the non-breeding season before it went extinct (see Table 1). Second, we filtered species’ range maps only by the pristine habitats of a cell (as opposed to filtering for both pristine and current habitats as for SR_obs_) and subtracted SR_obs_ from it. This second component hence accounts for the loss of species expected in response to habitat conversion in a cell. The final SR_ext_ was the sum of both components. We sum up these two components in order to account for any loss of species that might have occurred due to anthropogenic pressures other than land use, such as overharvesting.

The third SR map includes the subset of those species from SR_obs_ currently categorized as threatened in areas covered by national or regional Red Lists (SR_threat_; for more details see below/Table 1). We included threatened species to account for an extinction debt, i.e. to extinctions already triggered by extraction of biological production and/or habitat conversion, but not yet realized. We emphasize, however, that not all threatened species will necessarily go extinct over time. As a corollary, the inclusion of SR_threat_ will likely result in an overestimation of already realized and impending species loss in at least part of the cells. We also note that the threat status refers to the entire territory of the respective national/regional Red Lists and does not specify whether the population in a particular 20 x 20 km cell is threatened or not. Finally, national/ regional red lists are only available for parts of the globe and that SR_threat_ could hence only be computed for these parts.

National and regional (sub-national) red lists were provided by the National Red List project (<https://www.nationalredlist.org/>; 2018-06-29). These lists included 2972 mammal, 4654 bird and 2350 amphibian species and were available for 89 countries or regions. As each list operates with its own threat levels, we collated them to a binary threat status (threatened vs. not threatened, see Table S1.7 for an overview). Species from SR_obs_ were marked as species in SR_threat_ when a specific grid cell was both located within a nation/region where the species is marked as threatened and contained that species in SR_obs_. National and regional (and some supra-national) boundaries as named in the National Red List project were spatially assigned to grid cells via the standard ESRI sub-national boundary shape file (<https://www.arcgis.com/home/item.html?id=deb60dd7744048cd9ba4fe203881fd12>).

#### **1.2 Calculating HANPP components**

To calculate HANPP components we followed established procedures described in detail in Haberl et al. (2007) and Krausmann et al. (2013). We extrapolated HANPP_harv_ on cropland and forests based on harvest statistics from Food and Agriculture Organization of the United Nations (2020) Statistical Databases. (http://faostat.fao.org) by applying expansion factors that account for plant components not included in these statistics. On cropland, these expansion factors account for used and unused byproducts (based on harvest indices (Krausmann et al. 2013; Wirsenius 2000) and below-ground biomass (derived via root/shoot factors from Saugier et al. (2001)). For forestry, harvest volumes were expanded to include unreported fractions (i.e. bark, unrecovered fellings, and below-ground biomass) (Haberl et al. 2007; Krausmann et al. 2013). For livestock grazing, we calculated the biomass demand from grazing land as a function of per-head feed demand, separately for different animal species, and feed supply from other sources than grazing land (as in Krausmann et al. (2013)).

HANPP_luc_ was calculated as the difference between NPP_pot_ and the actual NPP of the currently prevailing vegetation, i.e. the productivity that remains after land use change has taken place (NPP_act_; by contrast, NPP_eco_ is what remains after both land use change and harvest have been accounted for). NPP_act_ for cropland was derived by expanding HANPP_harv_ with factors accounting for pre-harvest losses (based on Oerke et al. (1994)) dependent on fertilizer use per agricultural land (from FAO). Fallow cropland was assessed at the national level, assuming fallows to prevail when the sum of harvested areas from production statistics was smaller than the cropland area from FAO land-use statistics. NPP_act_ on fallow cropland was assumed to be 60% of NPP_pot_. NPP_act_ of natural grassland and forests was assumed to equal NPP_pot_, while for artificial grazing land (grazing land on potentially forested sites) it was assumed to be 80% of NPP_pot_ (Haberl et al. 2007). An additional reduction of productivity was assumed for degraded drylands (Zika and Erb 2009).

These national data were downscaled to the grid with land-use information for infrastructure areas, cropping, grazing and forestry (wilderness was excluded from harvest allocation). We used data from the HYDE database (Klein Goldewijk et al. 2017, https://doi.org/10.5194/essd-9-927-2017) to differentiate infrastructure, croplands, permanent pastures and rangelands.

High-resolution land-cover data (ESA-CCI land cover ESA (2017)) were used to identify closed (>40% tree cover) and open (15-40% tree cover) forests, and to separate other wooded lands from grazing land with few and no trees ( Bjelle et al. 2020, Semenchuk et al. 2022).

Cropland harvest was allocated to croplands following NPP_pot_ (from a modified run without water limitation on photosynthesis), fertilization (Nishina et al. 2017) and irrigation patterns (Klein Goldewijk et al. 2017), as in Kastner et al. (2022). Grazing harvest was allocated to pastures, rangelands and partly open forests based on the NPP available for grazing, favoring areas with high productivity by assigning a maximum grazing intensity of 40% at a level of accessible NPP of 20gC/m² and increasing this linearly to a maximum grazing intensity of 80% at 250gC/m². Forestry harvest (timber and wood fuel) was allocated to closed forests, parts of open forests and other wooded lands, following NPP_pot_ patterns, therefore depicting system-level effects of wood harvest (Haberl et al. 2007; Haberl et al. 2014).

## 2. Supplementary Tables and Figures

**Table S1.1.** Results for the species-energy-model for birds including adjusted partial R^2^ for each variable.

SER formula: (SR_birds_obs_ +SR_birds_extinct_) ~ NPP_pot_ + Realms + Mainland; family=quasiPoisson

| Birds | Estimate | Std. Error | t-value | p-value |  |
| --- | --- | --- | --- | --- | --- |
| Int.(NeoTropical) | 3.27 | 0.13 | 24.51 | < 2e-16 |  |
| NPP_pot_ | 1.5e-7 | 3.9e-9 | 38.12 | < 2e-16 | 44.38 |
| Australian | -0.12 | 0.03 | -3.76 | 1.7e-4 | 67.20 |
| AfroTropical | -0.86 | 0.05 | -18.49 | < 2e-16 |  |
| Oceanian | -0.88 | 0.06 | -13.92 | < 2e-16 |  |
| Oriental | -1.08 | 0.05 | -23.66 | < 2e-16 |  |
| Panamanian | -0.24 | 0.16 | -1.50 | 0.13 |  |
| Saharo-Arabian | -1.82 | 0.06 | -30.03 | < 2e-16 |  |
| Nearctic | -0.63 | 0.04 | -16.06 | < 2e-16 |  |
| Sino-Japanese | -1.83 | 0.06 | -31.37 | < 2e-16 |  |
| Palearctic | -0.55 | 0.04 | -13.07 | < 2e-16 |  |
| Mainland | 1.31 | 0.13 | 10.17 | 0.95 | 2.69 |

**Table S1.2.** Results for the species-energy-model for mammals including adjusted partial R^2^ for each variable.

SER formula: (SR_mammals_obs_+SR_mammals_extinct_) ~ NPP_pot_ + Realms + Mainland; family=quasiPoisson

| Mammals | Estimate | Std. Error | t-value | p-value |  |
| --- | --- | --- | --- | --- | --- |
| Int.(NeoTropical) | 1.97 | 0.11 | 18.18 | < 2e-16 |  |
| NPP_pot_ | 2.1e-7 | 3.1e-9 | 54.12 | < 2e-16 | 62.81 |
| Australian | -0.60 | 0.03 | -21.97 | < 2e-16 | 74.25 |
| AfroTropical | -0.49 | 0.03 | -14.23 | < 2e-16 |  |
| Oceanian | -1.76 | 0.07 | -25.15 | < 2e-16 |  |
| Oriental | -1.07 | 0.03 | -32.79 | < 2e-16 |  |
| Panamanian | -0.47 | 0.13 | -3.66 | 2.5e-4 |  |
| Saharo-Arabian | -0.81 | 0.04 | -21.70 | < 2e-16 |  |
| Nearctic | -0.33 | 0.03 | -11.33 | < 2e-16 |  |
| Sino-Japanese | -0.98 | 0.04 | -26.60 | < 2e-16 |  |
| Palearctic | -0.51 | 0.03 | -15.65 | < 2e-16 |  |
| Mainland | 1.38 | 0.01 | 13.18 | < 2e-16 | 3.54 |

**Table S1.3.** Results for the species-energy-model for amphibians including adjusted partial R^2^ for each variable.

SER formula: (SR_amphibians_obs_+SR_amphibians_extinct_) ~ NPP_pot_ + Realms + Mainland; family=quasiPoisson

| Amphibians | Estimate | Std. Error | t-value | p-value | Partial R^2^ |
| --- | --- | --- | --- | --- | --- |
| Int.(NeoTropical) | -13.22 | 251.2 | -0.05 | 0.95 |  |
| NPP_pot_ | 2.1e-7 | 5.8e-9 | 35.20 | < 2e-16 | 44.38 |
| Australian | -0.76 | 0.05 | -14.73 | < 2e-16 | 67.20 |
| AfroTropical | -1.77 | 0.08 | -20.90 | < 2e-16 |  |
| Oceanian | -2.11 | 0.13 | -16.68 | < 2e-16 |  |
| Oriental | -2.70 | 0.11 | -25.69 | < 2e-16 |  |
| Panamanian | -0.97 | 0.26 | -3.68 | 2.4e-4 |  |
| Saharo-Arabian | -3.54 | 0.17 | -20.41 | < 2e-16 |  |
| Nearctic | -1.78 | 0.07 | -24.04 | < 2e-16 |  |
| Sino-Japanese | -4.46 | 0.25 | -17.61 | < 2e-16 |  |
| Palearctic | -2.40 | 0.10 | -23.72 | < 2e-16 |  |
| Mainland | 15.32 | 251.2 | 0.06 | 0.95 | 2.69 |

**Table S1.4**. Attribution of Terrestrial Ecoregions of the World (as defined by Olson et al. (2001) and Dinerstein et al. (2017)) to the IUCN habitat classification system used for classifying species’ habitat affiliations (IUCN 2020).

| **Terrestrial Ecoregions of the World (Olson et al. 2001)**  **IUCN habitat classification** | **Tropical & Subtropical Moist Broadleaf Forests** | **Tropical & Subtropical Dry Broadleaf Forests** | **Tropical & Subtropical Coniferous Forests** | **Temperate Broadleaf & Mixed Forests** | **Temperate Conifer Forests** | **Boreal Forests/Taiga** | **Tropical & Subtropical Grasslands, Savannas & Shrublands** | **Temperate Grasslands, Savannas & Shrublands** | **Flooded Grasslands & Savannas** | **Montane Grasslands & Shrublands** | **Tundra** | **Mediterranean Forests, Woodlands & Scrub** | **Deserts & Xeric Shrublands** | **Mangroves** |
| --- | --- | --- | --- | --- | --- | --- | --- | --- | --- | --- | --- | --- | --- | --- |
| Forest - Boreal |  |  |  | x |  | x |  |  |  |  |  |  |  |  |
| Forest - Subarctic |  |  |  | x |  | x |  |  |  |  |  |  |  |  |
| Forest - Subantarctic |  |  |  | x |  | x |  |  |  |  |  |  |  |  |
| Forest - Temperate |  |  |  | x | x |  |  |  |  |  |  | x |  |  |
| Forest - Subtropical/Tropical Dry |  | x | x |  |  |  | x |  | x |  |  | x | x |  |
| Forest - Subtropical/Tropical Moist Lowland | x |  | x |  |  |  |  |  |  |  |  |  |  | x |
| Forest - Subtropical/Tropical Mangrove Vegetation Above High Tide Level | x | x |  |  |  |  |  |  |  |  |  |  |  | x |
| Forest - Subtropical/Tropical Swamp | x | x | x |  |  |  | x |  | x |  |  | x |  | x |
| Forest - Subtropical/Tropical Moist Montane | x |  | x |  |  |  |  |  |  |  |  |  |  |  |
| Savanna - Dry |  | x | x |  |  |  | x | x |  | x |  |  | x |  |
| Savanna - Moist | x |  | x |  |  |  | x | x | x | x |  |  |  |  |
| Shrubland - Subarctic |  |  |  | x |  | x |  |  |  | x | x |  |  |  |
| Shrubland - Subantarctic |  |  |  | x |  | x |  |  |  | x | x |  |  |  |
| Shrubland - Boreal |  |  |  |  |  | x |  |  |  | x |  |  |  |  |
| Shrubland - Temperate |  |  |  | x | x |  |  | x |  | x |  | x | x |  |
| Shrubland - Subtropical/Tropical Dry |  | x | x |  |  |  | x |  |  | x |  | x | x |  |
| Shrubland - Subtropical/Tropical Moist | x |  | x |  |  |  | x |  | x | x |  |  |  | x |
| Shrubland - Subtropical/Tropical High Altitude | x | x | x |  |  |  | x |  |  | x |  |  | x |  |
| Shrubland - Mediterranean-type Shrubby Vegetation |  |  |  |  |  |  |  |  |  | x |  | x | x |  |
| Grassland - Tundra |  |  |  |  |  |  |  |  |  | x | x |  |  |  |
| Grassland - Subarctic |  |  |  |  |  |  |  |  |  |  | x |  |  |  |
| Grassland - Subantarctic |  |  |  |  |  |  |  |  |  |  | x |  |  |  |
| Grassland - Temperate |  |  |  |  |  |  |  | x |  | x |  |  |  |  |
| Grassland - Subtropical/Tropical Dry |  |  |  |  |  |  | x |  | x |  |  |  | x |  |
| Grassland - Subtropical/Tropical Seasonally Wet/Flooded |  |  |  |  |  |  |  |  | x |  |  |  |  |  |
| Grassland - Subtropical/Tropical High Altitude |  |  |  |  |  |  | x |  |  | x |  |  | x |  |
| Wetlands (inland) - Permanent Rivers/Streams/Creeks (includes waterfalls) | x | x | x | x | x | x | x | x | x | x | x | x | x | x |
| Wetlands (inland) - Seasonal/Intermittent/Irregular Rivers/Streams/Creeks | x | x | x | x | x | x | x | x | x | x | x | x | x | x |
| Wetlands (inland) - Shrub Dominated Wetlands | x | x | x | x | x | x | x | x | x | x | x | x | x | x |
| Wetlands (inland) - Bogs, Marshes, Swamps, Fens, Peatlands | x | x | x | x | x | x | x | x | x | x | x | x | x | x |
| Wetlands (inland) - Permanent Freshwater Lakes (over 8ha) |  |  |  |  |  |  |  |  |  |  |  |  |  |  |
| Wetlands (inland) - Seasonal/Intermittent Freshwater Lakes (over 8ha) |  |  |  |  |  |  |  |  |  |  |  |  |  |  |
| Wetlands (inland) - Permanent Freshwater Marshes/Pools (under 8ha) | x | x | x | x | x | x | x | x | x | x | x | x | x | x |
| Wetlands (inland) - Seasonal/Intermittent Freshwater Marshes/Pools (under 8ha) | x | x | x | x | x | x | x | x | x | x | x | x | x | x |
| Wetlands (inland) - Freshwater Springs and Oases | x | x | x | x | x | x | x | x | x | x | x | x | x | x |
| Wetlands (inland) - Tundra Wetlands (incl. pools and temporary waters from snowmelt) |  |  |  |  |  |  |  |  |  |  | x |  |  |  |
| Wetlands (inland) - Alpine Wetlands (includes temporary waters from snowmelt) |  |  |  |  |  |  |  |  |  | x | x |  |  |  |
| Wetlands (inland) – Geothermal wetlands |  |  |  |  |  |  |  |  |  |  |  |  |  |  |
| Wetlands (inland) – Permanent inland deltas |  |  |  |  |  |  |  |  |  |  |  |  |  |  |
| Wetlands (inland) – Permanent saline, brackish or alkaline lakes |  |  |  |  |  |  |  |  |  |  |  |  |  |  |
| Wetlands (inland) – Seasonal/intermittent saline, brackish or alkaline lakes and flats | x | x | x | x | x | x | x | x | x |  |  | x | x | x |
| Wetlands (inland) – Permanent saline, brackish or alkaline marshes/pools | x | x | x | x | x | x | x | x | x |  |  | x | x | x |
| Wetlands (inland) – Seasonal/intermittent saline, brackish or alkaline marshes/pools | x | x | x | x | x | x | x | x | x |  |  | x | x | x |
| Wetlands (inland) – Karst and other subterranean hydrological systems (inland) |  |  |  |  |  |  |  |  |  |  |  |  |  |  |
| Desert – Hot |  |  |  |  |  |  | x |  |  |  |  |  | x |  |
| Desert – Temperate |  |  |  |  |  |  |  | x |  |  |  |  | x |  |
| Desert – Cold |  |  |  |  |  |  |  |  |  |  | x |  | x |  |
| Arable Land | x | x | x | x | x | x | x | x | x | x | x | x | x | x |
| Pastureland | x | x | x | x | x | x | x | x | x | x | x | x | x | x |
| Plantations | x | x | x | x | x | x | x | x | x | x |  | x | x | x |
| Rural Gardens | x | x | x | x | x | x | x | x | x | x |  | x | x | x |
| Urban Areas | x | x | x | x | x | x | x | x | x | x | x | x | x | x |
| Subtropical/Tropical Heavily Degraded Former Forest | x | x | x |  |  |  | x |  | x | x |  | x | x |  |
| Introduced Vegetation |  |  |  |  |  |  |  |  |  |  |  |  |  |  |
| Rocky areas (eg. inland cliffs, mountain peaks) | x | x | x | x | x | x | x | x | x | x | x | x | x | x |

**Table S1.5.** Attribution of ESA CCI land cover types (ESA 2017) to the IUCN habitat classification system used for classifying species’ habitat affiliations (IUCN 2020).

| **ESA CCI land cover type**  **IUCN habitat**  **classification** | **Cropland, rainfed** | **Cropland, irrigated or post-flooding** | **Mosaic cropland (>50%) / natural vegetation (tree, shrub, herbaceous cover) (<50%)** | **Mosaic natural vegetation (tree, shrub, herbaceous cover) (>50%) / cropland (<50%) /** | **Tree cover, broadleaved, evergreen, closed to open (<15%)** | **Tree cover, broadleaved, deciduous, closed to open (<15%)** | **Tree cover, needleleaved, evergreen, closed to open (<15%)** | **Tree cover, needleleaved, deciduous, closed to open (<15%)** | **Tree cover, mixed leaf type (broadleaved and needleleaved)** | **Mosaic tree and shrub (>50%) / herbaceous cover (<50%)** | **Mosaic herbaceous cover (>50%) / tree and shrub (<50%)** | **Shrubland** | **Grassland** | **Lichens and mosses** | **Sparse vegetation (tree, shrub, herbaceous cover) (<15%)** | **Tree cover, flooded, fresh or brakish water** | **Tree cover, flooded, saline water** | **Shrub or herbaceous cover, flooded, fresh/saline/brakish water** | **Urban areas** | **Bare areas** |
| --- | --- | --- | --- | --- | --- | --- | --- | --- | --- | --- | --- | --- | --- | --- | --- | --- | --- | --- | --- | --- |
| Forest – Boreal |  |  | x | x |  | x | x | x | x | x | x |  |  |  |  |  |  |  |  |  |
| Forest – Subarctic |  |  |  |  |  | x | x |  | x | x | x |  |  |  |  |  |  |  |  |  |
| Forest – Subantarctic |  |  |  |  |  | x | x |  | x | x | x |  |  |  |  |  |  |  |  |  |
| Forest – Temperate |  |  | x | x |  | x | x | x | x | x | x |  |  |  |  |  |  |  |  |  |
| Forest – Subtropical/tropical dry |  |  | x | x | x | x | x |  | x | x | x |  |  |  |  |  |  |  |  |  |
| Forest – Subtropical/tropical moist lowland |  |  | x | x | x |  |  |  |  | x | x |  |  |  |  |  |  |  |  |  |
| Forest – Subtropical/tropical mangrove vegetation above high tide level |  |  |  |  | x |  |  |  |  | x | x |  |  |  |  | x | x |  |  |  |
| Forest – Subtropical/tropical swamp |  |  |  |  | x |  |  |  |  | x | x |  |  |  |  | x | x |  |  |  |
| Forest – Subtropical/tropical moist montane |  |  | x | x | x | x | x |  | x | x | x |  |  |  |  |  |  |  |  |  |
| Savanna - Dry |  |  | x | x |  |  |  |  |  |  |  |  |  |  |  |  |  |  |  |  |
| Savanna - Moist |  |  | x | x |  |  |  |  |  | x | x |  | x |  |  |  |  |  |  |  |
| Shrubland – Subarctic |  |  |  |  |  |  |  |  |  | x | x | x |  |  |  |  |  |  |  |  |
| Shrubland – Subantarctic |  |  |  |  |  |  |  |  |  | x | x | x |  |  |  |  |  |  |  |  |
| Shrubland – Boreal |  |  | x | x |  |  |  |  |  | x | x | x |  |  |  |  |  |  |  |  |
| Shrubland –Temperate |  |  | x | x |  |  |  |  |  | x | x | x |  |  |  |  |  |  |  |  |
| Shrubland – Subtropical/tropical dry |  |  | x | x |  |  |  |  |  | x | x | x |  |  |  |  |  |  |  |  |
| Shrubland – Subtropical/tropical moist |  |  | x | x |  |  |  |  |  | x | x | x |  |  |  |  |  |  |  |  |
| Shrubland – Subtropical/tropical high altitude |  |  | x | x |  |  |  |  |  | x | x | x |  |  |  |  |  |  |  |  |
| Shrubland – Mediterranean- type shrubby vegetation |  |  | x | x |  |  |  |  |  | x | x | x |  |  |  |  |  |  |  |  |
| Grassland – Tundra |  |  |  |  |  |  |  |  |  | x | x |  | x |  |  |  |  |  |  |  |
| Grassland – Subarctic |  |  |  |  |  |  |  |  |  | x | x |  | x |  |  |  |  |  |  |  |
| Grassland – Subantarctic |  |  |  |  |  |  |  |  |  | x | x |  | x |  |  |  |  |  |  |  |
| Grassland – Temperate |  |  | x | x |  |  |  |  |  | x | x |  | x |  |  |  |  |  |  |  |
| Grassland – Subtropical/tropical dry |  |  | x | x |  |  |  |  |  | x | x |  | x |  |  |  |  |  |  |  |
| Grassland – Subtropical/tropical seasonally wet/flooded |  |  | x | x |  |  |  |  |  | x | x |  | x |  |  |  |  |  |  |  |
| Grassland – Subtropical/tropical high altitude |  |  | x | x |  |  |  |  |  | x | x |  | x |  |  |  |  |  |  |  |
| Wetlands (inland) – Permanent rivers/streams/creeks (includes waterfalls) |  |  |  |  |  |  |  |  |  |  |  |  |  |  |  |  |  |  |  |  |
| Wetlands (inland) – Seasonal/intermittent/irregular rivers/streams/creeks |  |  |  |  |  |  |  |  |  |  |  |  |  |  |  |  |  |  |  |  |
| Wetlands (inland) – Shrub dominated wetlands |  |  | x | x |  |  |  |  |  | x | x | x |  |  |  |  |  | x |  |  |
| Wetlands (inland) – Bogs, marshes, swamps, fens, peatlands |  |  | x | x |  |  |  |  |  | x | x |  | x | x |  | x |  | x |  |  |
| Wetlands (inland) – Permanent freshwater lakes (over 8 ha) |  |  |  |  |  |  |  |  |  |  |  |  |  |  |  |  |  |  |  |  |
| Wetlands (inland) – Seasonal/intermittent freshwater lakes (over 8 ha) |  |  |  |  |  |  |  |  |  |  |  |  |  |  |  |  |  |  |  |  |
| Wetlands (inland) – Permanent freshwater marshes/pools (under 8 ha) |  |  | x | x |  |  |  |  |  | x | x |  | x |  |  |  |  |  |  |  |
| Wetlands (inland) – Seasonal/intermittent freshwater marshes/pools (under 8 ha) |  |  | x | x |  |  |  |  |  | x | x |  | x |  |  |  |  |  |  |  |
| Wetlands (inland) – Freshwater springs and oases |  |  | x | x | x | x |  |  |  | x | x | x | x |  |  |  |  |  |  |  |
| Wetlands (inland) – Tundra wetlands (inc. pools and temporary waters from snowmelt) |  |  |  |  |  |  |  |  |  | x | x |  | x | x |  |  |  |  |  |  |
| Wetlands (inland) – Alpine wetlands (inc. temporary waters from snowmelt) |  |  |  |  |  |  |  |  |  | x | x |  | x | x |  |  |  |  |  |  |
| Wetlands (inland) – Geothermal wetlands |  |  |  |  |  |  |  |  |  |  |  |  | x | x |  |  |  |  |  |  |
| Wetlands (inland) – Permanent inland deltas |  |  |  |  | x | x | x | x | x | x | x | x | x |  |  | x |  | x |  |  |
| Wetlands (inland) – Permanent saline, brackish or alkaline lakes |  |  |  |  |  |  |  |  |  |  |  |  |  |  |  | x | x | x |  |  |
| Wetlands (inland) – Seasonal/intermittent saline, brackish or alkaline lakes and flats |  |  |  |  |  |  |  |  |  |  |  |  |  |  |  | x | x | x |  |  |
| Wetlands (inland) – Permanent saline, brackish or alkaline marshes/pools |  |  |  |  |  |  |  |  |  |  |  |  | x |  |  | x | x | x |  |  |
| Wetlands (inland) – Seasonal/intermittent saline, brackish or alkaline marshes/pools |  |  |  |  |  |  |  |  |  |  |  |  | x |  |  | x | x | x |  |  |
| Wetlands (inland) – Karst and other subterranean hydrological systems (inland) |  |  |  |  |  |  |  |  |  |  |  |  |  |  |  |  |  |  |  |  |
| Desert – Hot |  |  |  |  |  |  |  |  |  |  |  |  |  |  | x |  |  |  |  | x |
| Desert – Temperate |  |  |  |  |  |  |  |  |  |  |  |  |  |  | x |  |  |  |  | x |
| Desert – Cold |  |  |  |  |  |  |  |  |  |  |  |  |  |  | x |  |  |  |  | x |
| Arable Land | x | x | x | x |  |  |  |  |  |  |  |  |  |  |  |  |  |  |  |  |
| Pastureland |  |  | x | x |  |  |  |  |  | x | x |  | x |  |  |  |  |  |  |  |
| Plantations |  |  | x | x | x | x | x | x | x | x |  |  |  |  |  |  |  |  |  |  |
| Rural Gardens |  |  |  |  | x | x | x | x | x |  |  |  | x |  |  |  |  |  |  |  |
| Urban Areas |  |  |  |  |  |  |  |  |  |  |  |  |  |  | x |  |  |  | x |  |
| Subtropical/Tropical Heavily Degraded Former Forest |  |  | x | x | x | x |  |  |  | x | x | x |  |  |  |  |  |  |  |  |
| Introduced Vegetation |  |  | x | x |  |  |  |  |  | x | x |  |  |  |  |  |  |  |  |  |
| Rocky areas (e.g.. inland cliffs, mountain peaks) |  |  | x | x | x | x | x | x | x | x | x | x | x | x | x |  |  |  |  | x |

**Table S1.6.** Number of wilderness cells per zoogeographical realm as defined by Holt et al. (2013). Due to a lack of wilderness areas, the Madagascan realm was treated as no data zone in our analysis.

|  | Realms | Number of  wilderness cells |
| --- | --- | --- |
| 1 | NeoTropical | 7,524 |
| 2 | Australian | 6,827 |
| 3 | AfroTropical | 6,745 |
| 4 | Madagascan | - |
| 5 | Oceanian | 7 |
| 6 | Oriental | 156 |
| 7 | Panamanian | 9 |
| 8 | Saharo-Arabian | 5,803 |
| 9 | Nearctic | 11,482 |
| 10 | Sino-Japanese | 1,615 |
| 11 | Palearctic | 24,752 |

**Table S1.7** Table showing the translation of the multitude of national Red List threat levels to a binary classification of threatened or not threatened.

| **Regions** | **Regional status** | **Threatened** |
| --- | --- | --- |
| Afghanistan | Not Evaluated | No |
| Afghanistan, Argentina, Austria, Bhutan, Croatia, Czech Republic, India, Mexico, Mongolia, Myanmar, NEPAL, Pakistan, South Africa, Lesotho, Swaziland, South Asia, Sri Lanka, Sweden, Switzerland, United Kingdom | Not Evaluated | No |
| Albania | Lower Risk/Conservation Dependent | No |
| Albania | Lower Risk/Least Concern | No |
| Albania | Lower Risk/Near Threatened | No |
| Albania, Argentina, Australia Victoria, Austria, Bangladesh, Belgium Flanders, Bhutan, Brazil, Canada, Carpathian Mountains, China, Colombia, Croatia, Czech Republic, Ecuador, Estonia, Finland, France Adélie Land, France continental, France French Southern Territories, France Guadeloupe, France mainland and Corsica, France Mayotte, France Réunion, France Scattered Islands, India, Israel, Italy, Japan, Mongolia, Nepal, Norway, Pakistan, Poland, Republic of Korea, South Africa, Lesotho, Swaziland, South Asia, Sri Lanka, Sweden, Switzerland, Uganda, Uruguay, Venezuela | Data Deficient | No |
| Albania, Argentina, Australia, Australia Victoria, Austria, Belgium Flanders, Bolivia, Brazil, Bulgaria, Canada, China, Colombia, Croatia, Cuba, Czech Republic, Dominican Republic, Ecuador, Estonia, Finland, France Adélie Land, France continental, France French Southern Territories, France Guadeloupe, France mainland and Corsica, France Mayotte, France Réunion, Greece, Iceland, India, Italy, Japan, Mediterranean Basin, Mexico, Nepal, Nicaragua, Norway, Republic of Korea, South Africa, Lesotho, Swaziland, Sri Lanka, Sweden, Uganda, Uruguay | Critically Endangered | Yes |
| Albania, Argentina, Australia, Australia Victoria, Austria, Belgium Flanders, Bolivia, Brazil, Bulgaria, Canada, China, Colombia, Croatia, Cuba, Dominican Republic, Ecuador, Estonia, Finland, France Adélie Land, France continental, France French Southern Territories, France Guadeloupe, France mainland and Corsica, France Mayotte, France Réunion, France Scattered Islands, Greece, Iceland, Ireland, Italy, Japan, Lithuania, Mediterranean Basin, Mexico, Nepal, Nicaragua, Norway, Republic of Korea, South Africa, Lesotho, Swaziland, Sri Lanka, Sweden, Uganda, Uruguay | Endangered | Yes |
| Albania, Argentina, Australia, Australia Victoria, Austria, Belgium Flanders, Bolivia, Brazil, Bulgaria, Canada, Chile, China, Colombia, Croatia, Cuba, Czech republic, Dominican Republic, Ecuador, Estonia, Finland, France Adélie Land, France continental, France French Southern Territories, France Guadeloupe, France mainland and Corsica, France Mayotte, France Réunion, France Scattered Islands, Greece, Iceland, Italy,  Japan, Lithuania, Mediterranean Basin, Mexico, Nepal, Nicaragua, Norway, Republic of Korea, South Africa, Lesotho, Swaziland, Sri Lanka, Sweden, Uganda, United Kingdom, Uruguay, Venezuela | Vulnerable | Yes |
| Albania, Argentina, Australia, Australia Victoria, Brazil, Bulgaria, Canada, Carpathian Mountains, China, Colombia, Czech Republic, Ecuador, France French Southern Territories, France Guadeloupe, France Réunion, Honduras, Iceland, Israel, New Zealand, Poland, Republic of Korea, South Africa, Lesotho, Swaziland, Sri Lanka, Venezuela | Extinct | Yes |
| Andorra | E | Yes |
| Andorra | I | No |
| Andorra | S | No |
| Andorra | V | Yes |
| Andorra, Luxembourg | R | No |
| Argentina | DI | No |
| Argentina | En Peligro Critico/ Critically Endangered; A1acd,B12abcde,C12ab,E | Yes |
| Argentina | En Peligro Critico/ Critically Endangered; A1d,B2d | Yes |
| Argentina | En Peligro Critico/ Critically Endangered; B1 | Yes |
| Argentina | En Peligro/ Endangered; A1acd,B12abc,C2a | Yes |
| Argentina | En Peligro/ Endangered; A1acd,B12abcd,C12a,D,E | Yes |
| Argentina | En Peligro/ Endangered; A1acd,B12ad,C2a | Yes |
| Argentina | En Peligro/ Endangered; A1bc,A2acde,B1,B2abcde,C1,C2a | Yes |
| Argentina | En Peligro/ Endangered; A1C,A2acde,C2a | Yes |
| Argentina | En Peligro/ Endangered; A1cd,A2cde,B1,B2,C1 | Yes |
| Argentina | En Peligro/ Endangered; B1 | Yes |
| Argentina | En Peligro/ Endangered; B1,2c | Yes |
| Argentina | En Peligro/ Endangered; C1 | Yes |
| Argentina | EP | Yes |
| Argentina | RBdc | No |
| Argentina | RBpm | No |
| Argentina | RBpv | No |
| Argentina | Vulnerable; A1acd | Yes |
| Argentina | Vulnerable; A1acd,C12b | Yes |
| Argentina | Vulnerable; A1acde,B12abcd,C2a,E | Yes |
| Argentina | Vulnerable; A1bc,B1,B2c | Yes |
| Argentina | Vulnerable; A1cde | Yes |
| Argentina | Vulnerable; A1d | Yes |
| Argentina | Vulnerable; A2c,B1,2c | Yes |
| Argentina | Vulnerable; A2c,B2c | Yes |
| Argentina | Vulnerable; B1 | Yes |
| Argentina | Vulnerable; B1,2c | Yes |
| Argentina | Vulnerable; B2a,B3a,C1 | Yes |
| Argentina | Vulnerable; B2c | Yes |
| Argentina, Australia Victoria, Austria, Bangladesh, Belgium Flanders, Bhutan, Bolivia, Brazil, China, Colombia, Croatia, Cuba, Czech Republic, Ecuador, Estonia, Finland, France continental, France French Southern Territories, France Guadeloupe, France mainland and Corsica, France Mayotte, France Réunion, France Scattered Islands, India, Israel, Italy, Japan, Mediterranean Basin, Mongolia, Nepal, Norway, Pakistan, Poland, Republic of Korea, South Africa, South Africa, Lesotho, Swaziland, South Asia, Sri Lanka, Sweden, Switzerland, Uganda, Uruguay, Venezuela | Near Threatened | No |
| Argentina, Austria, Bangladesh, Belgium Flanders, Bhutan, Canada, China, Colombia, Croatia, Czech Republic, Ecuador, Estonia, France Adélie Land, France continental, France French Southern Territories, France Guadeloupe, France mainland and Corsica, France Mayotte, France Réunion, France Scattered Islands, India, Ireland, Israel, Italy, Maldives, Mediterranean Basin, Mexico, Mongolia, Nepal, Pakistan, Poland, Republic of Korea, South Africa, Lesotho, Swaziland, South Asia, Sri Lanka, Sweden, Switzerland, Uganda, Uruguay, Venezuela | Least Concern | No |
| Argentina, Austria, Belgium Wallonia, Bulgaria, Luxembourg | VU | Yes |
| Argentina, Bulgaria, Luxembourg | EX | Yes |
| Argentina, Finland | Vulnerable; A1ab | Yes |
| Australia Victoria, Austria, Belgium Flanders, Brazil, China, Croatia, Czech Republic, Estonia, Finland, France continental, France French Southern Territories, France Guadeloupe, France mainland and Corsica, France Réunion, France Scattered Islands, Greece, Israel, Italy, Mongolia, Nepal, Norway, Republic of Korea, Sweden, Switzerland, Uganda, Uruguay | Regionally Extinct | Yes |
| Australia Victoria, Brazil | Extinct in the Wild | Yes |
| Austria | DD | No |
| Austria | NE | No |
| Austria, Belgium Wallonia | LC | No |
| Austria, Belgium Wallonia | RE | Yes |
| Austria, Belgium Wallonia, Bulgaria, Luxembourg | CR | Yes |
| Austria, Belgium Wallonia, Bulgaria, Luxembourg | EN | Yes |
| Austria, Belgium Wallonia, Luxembourg | NT | No |
| Bangladesh | Critically Endangered; A2c; C1+2a(i); D | Yes |
| Bangladesh | Critically Endangered; B2ab(i,ii,iii,iv,v); C2a(i) | Yes |
| Bangladesh | Critically Endangered; C1+2a(i) | Yes |
| Bangladesh | Endangered; A2acd | Yes |
| Bangladesh | Endangered; B1ab(ii,iii,v)+2ab(ii,iii,v) | Yes |
| Bangladesh | Endangered; B1ab(iii); D1 | Yes |
| Bangladesh | Locally Extinct | Yes |
| Bangladesh | Vulnerable Downgraded to Near Threatened; D2 | Yes |
| Bangladesh, Bhutan, India, Nepal, Pakistan | Vulnerable. Downgraded to Near Threatened; B1ab(iii)+2ab(iii) | Yes |
| Bangladesh, Bhutan, Nepal | Endangered downgraded to Near Threatened; B2ab(iii) | Yes |
| Bangladesh, Bhutan, Nepal | Vulnerable Downgraded to Near Threatened; B1ab(iii)+2ab(iii) | Yes |
| Bangladesh, India, Nepal, Pakistan, South Asia | Vulnerable; A2c+3c+4c | Yes |
| Bangladesh, India, Nepal, South Asia | Vulnerable; B2ab(iii) | Yes |
| Bangladesh, Nepal | Endangered Downgraded to Vulnerable; B1ab(ii,iii)+2ab(ii,iii) | Yes |
| Bangladesh, Nepal | Vulnerable downgraded to Near Threatened; B1ab(ii,iii)+2ab(ii,iii) | Yes |
| Bangladesh, South Asia | Critically Endangered; C2a(i) | Yes |
| Bangladesh, Sweden | Critically Endangered; C2a(i); D | Yes |
| Bangladesh, Venezuela | Endangered; C2a(ii) | Yes |
| Belgium Flanders | Categorie ? (Datadeficient) | No |
| Belgium Flanders | Categorie 0 (Extinct) | Yes |
| Belgium Flanders | Categorie 1 (Critically endangered) | Yes |
| Belgium Flanders | Categorie 2 (Endangered) | Yes |
| Belgium Flanders | Categorie 3 (Vulnerable) | Yes |
| Belgium Flanders | Categorie A (Near threatened) | No |
| Belgium Flanders | Categorie N. (Not threatened) | No |
| Belgium Flanders | Categorie Z (Rare) | No |
| Bhutan | Critically Endangered downgraded to Endangered; C2a(i); D | Yes |
| Bhutan | Endangered; A2c+3c+4c; B1ab(ii,iii) | Yes |
| Bhutan, India, Nepal | Endangered Downgraded to Vulnerable; B1ab(iii)+2ab(iii) | Yes |
| Bhutan, India, South Asia | Endangered; C2a(i) | Yes |
| Bhutan, Nepal | Endangered Downgraded to Vulnerable; B2ab(ii,iii) | Yes |
| Bhutan, Nepal | Endangered; A2c+3c+4c; 2ab(ii,iii) | Yes |
| Bhutan, South Asia | Endangered; B1ab(i,ii,iii,iv,v); C1+2a | Yes |
| Bolivia | Vulerable | Yes |
| Canada | Data Deficient; Données insuffisantes | No |
| Canada | Endangered; En voie de disparition; A1abc; C2a; D1 | Yes |
| Canada | Endangered; En voie de disparition; A1ac; B1+2de+3d; C2b; D1 | Yes |
| Canada | Endangered; En voie de disparition; A1acde; B1+2abcd; C2a; D1 | Yes |
| Canada | Endangered; En voie de disparition; A1ad; D1 | Yes |
| Canada | Endangered; En voie de disparition; A1bd; D1 | Yes |
| Canada | Endangered; En voie de disparition; A1c+2c; B1+2c | Yes |
| Canada | Endangered; En voie de disparition; A1d; D1 | Yes |
| Canada | Endangered; En voie de disparition; A2a | Yes |
| Canada | Endangered; En voie de disparition; A2a; C1 | Yes |
| Canada | Endangered; En voie de disparition; A2a; C2a(i); D1 | Yes |
| Canada | Endangered; En voie de disparition; A2a; D1 | Yes |
| Canada | Endangered; En voie de disparition; A2ac; B2ab(i,ii,iii,iv,v); C1+2a(i); D1; E | Yes |
| Canada | Endangered; En voie de disparition; A2b | Yes |
| Canada | Endangered; En voie de disparition; A2b; C1 | Yes |
| Canada | Endangered; En voie de disparition; A2b; C1+2a(i) | Yes |
| Canada | Endangered; En voie de disparition; A2b; C2a(i); D1 | Yes |
| Canada | Endangered; En voie de disparition; A2bc; C1 | Yes |
| Canada | Endangered; En voie de disparition; A2bc; C2a(i) | Yes |
| Canada | Endangered; En voie de disparition; A2d; C1; E | Yes |
| Canada | Endangered; En voie de disparition; A3c; B2ab(iii); C2a(i) | Yes |
| Canada | Endangered; En voie de disparition; A3e+4ae; C1+2a(i) | Yes |
| Canada | Endangered; En voie de disparition; A4c; C1 | Yes |
| Canada | Endangered; En voie de disparition; B1+2abcde; C2b; D1 | Yes |
| Canada | Endangered; En voie de disparition; B1+2c; D1 | Yes |
| Canada | Endangered; En voie de disparition; B1ab(i,ii,iii)+2ab(i,ii,iii); C2a(i,ii); D1 | Yes |
| Canada | Endangered; En voie de disparition; B1ab(i,ii,iii,iv,v)+2ab(i,ii,iii,iv,v); C1+2a(i); D1 | Yes |
| Canada | Endangered; En voie de disparition; B1ab(i,ii,iii,iv,v)+2ab(i,ii,iii,iv,v); D1 | Yes |
| Canada | Endangered; En voie de disparition; B1ab(i,iii) | Yes |
| Canada | Endangered; En voie de disparition; B1ab(ii,iii,iv)+2ab(ii,iii,iv) | Yes |
| Canada | Endangered; En voie de disparition; B1ab(iii)+2ab(iii); C2a(i,ii); D1 | Yes |
| Canada | Endangered; En voie de disparition; B1ab(iii)+2ab(iii)c(iv) | Yes |
| Canada | Endangered; En voie de disparition; B1ab(iii,v)+2ab(iii,v) | Yes |
| Canada | Endangered; En voie de disparition; B1ac(iv)+2ac(iv); D1 | Yes |
| Canada | Endangered; En voie de disparition; B2ab(ii,iii,iv) | Yes |
| Canada | Endangered; En voie de disparition; C1 | Yes |
| Canada | Endangered; En voie de disparition; C1; D1 | Yes |
| Canada | Endangered; En voie de disparition; C2a | Yes |
| Canada | Endangered; En voie de disparition; C2a(i,ii); D1 | Yes |
| Canada | Endangered; En voie de disparition; C2a; D1 | Yes |
| Canada | Endangered; En voie de disparition; C2b; D1 | Yes |
| Canada | Endangered; En voie de disparition; C2b; E | Yes |
| Canada | Endangered; En voie de disparition; D1 | Yes |
| Canada | Extinct; Disparue | Yes |
| Canada | Extirpated; Disparue du pays | Yes |
| Canada | Not at Risk; Non en péril | No |
| Canada | Special Concern; Préoccupante | Yes |
| Canada | Special Concern; Préoccupante; (D2 -Threatened) | Yes |
| Canada | Special Concern; Préoccupante; D2 -Threatened | Yes |
| Canada | Threatened; Menacée | Yes |
| Canada | Threatened; Menacée; A1acd; D1 | Yes |
| Canada | Threatened; Menacée; A1bc | Yes |
| Canada | Threatened; Menacée; A1d | Yes |
| Canada | Threatened; Menacée; A2a | Yes |
| Canada | Threatened; Menacée; A2b | Yes |
| Canada | Threatened; Menacée; A2bc | Yes |
| Canada | Threatened; Menacée; A2c | Yes |
| Canada | Threatened; Menacée; A2e | Yes |
| Canada | Threatened; Menacée; A4ac | Yes |
| Canada | Threatened; Menacée; B1+2bc; D2 | Yes |
| Canada | Threatened; Menacée; B1+2c+3d; D1 | Yes |
| Canada | Threatened; Menacée; B1ab(ii,iii,v)+B2ab(ii,iii,v); D2 | Yes |
| Canada | Threatened; Menacée; B2ab(ii,iii)c(iv) | Yes |
| Canada | Threatened; Menacée; B2c+3cd; D2; E | Yes |
| Canada | Threatened; Menacée; C1 | Yes |
| Canada | Threatened; Menacée; C2b | Yes |
| Canada | Threatened; Menacée; D1 | Yes |
| Canada | Threatened; Menacée; D1+2 | Yes |
| Canada | Threatened; Menacée; D2 | Yes |
| Carpathian Mountains, Colombia, Croatia, India, Japan, Nepal, Pakistan, Poland, South Africa, Sri Lanka | Vulnerable: criteria unknown | Yes |
| Carpathian Mountains, Colombia, Croatia, Japan, Poland | Critically Endangered: criteria unknown | Yes |
| Carpathian Mountains, Colombia, Croatia, Japan, Poland, South Africa | Endangered: criteria unknown | Yes |
| Chile | En Peligro | Yes |
| Chile | En Peligro en las Regiones de Atacama y Coquimbo. Vulnerable en el resto del país | Yes |
| Chile | En Peligro y Rara | Yes |
| Chile | Insuficientemente Conocida | No |
| Chile | Insuficientemente Conocida y Rara | No |
| Chile | Rara | No |
| China | Regionaly Extinct | Yes |
| Colombia, Croatia, South Africa, Lesotho, Swaziland, Sri Lanka | Critically Endangered (Possibly Extinct) | Yes |
| Croatia | Not Available | No |
| Ecuador, Mexico | Neat Threatened | No |
| Estonia | Care demanding | Yes |
| Estonia | Extinct or probably extinct | Yes |
| Estonia | Vulnerab;e | Yes |
| Estonia, France Adélie Land, France continental, France French Southern Territories, France Guadeloupe, France mainland and Corsica, France Mayotte, France Réunion, France Scattered Islands, Italy, Sweden, Uganda, Uruguay | Not Applicable | No |
| Estonia, Lithuania, South Africa | Rare | No |
| Finland | Critically Endangered; A1a, C1+2b, D1 | Yes |
| Finland | Critically Endangered; A1ac, B1+2cde, D1 | Yes |
| Finland | Critically Endangered; C2a | Yes |
| Finland | Critically Endangered; C2a, D1 | Yes |
| Finland | Endangered; (CR D1) | Yes |
| Finland | Endangered; C2a, D1 | Yes |
| Finland | Near Threatened; (VU D1) | No |
| Finland | Vulnerable; (CR D1) | Yes |
| Finland | Vulnerable; (EN D1) | Yes |
| Finland | Vulnerable; A1ab, C1 | Yes |
| Finland | Vulnerable; A1ab+2ab | Yes |
| Finland | Vulnerable; A1abc | Yes |
| Finland | Vulnerable; A1bc, C1 | Yes |
| Finland | Vulnerable; B1+2c, C2a | Yes |
| Finland | Vulnerable; C1+2a | Yes |
| Finland, India, Israel, Nepal, Norway, Pakistan, South Asia, Sweden, Switzerland | Vulnerable; D1 | Yes |
| Finland, Israel | Vulnerable; A1ac | Yes |
| Finland, Norway | Endangered; D1 | Yes |
| Finland, Norway, Sweden, Switzerland, Venezuela | Vulnerable; C1 | Yes |
| Finland, Norway, Venezuela | Critically Endangered; D1 | Yes |
| Finland, Switzerland | Near Threatened; D1 | No |
| Germany Baden-Wuerttemberg | Threatened migratory species (Gefährdete wandernde Tierart) | Yes |
| Germany Baden-Wuerttemberg, Germany Brandenburg | Currently not threatened (Zur Zeit nicht gefährdet) | No |
| Germany Bayern | Extremely rare or restricted (Extrem selten oder geografische Restriktion) | No |
| Germany Bayern, Germany Berlin | Data deficient (Daten defizitär) | No |
| Germany Bayern, Germany Hessen, Germany Nordrhein-Westfalen | Not threatened (Ungefährdet) | No |
| Germany Bayern, Germany Nordrhein-Westfalen | Not Assessed (Nicht bewertet) | No |
| Germany Berlin | Cautionary reaction to be adopted(Gefahrdung anzunehmen) | Yes |
| Germany Berlin | Extinct or missing (Ausgestorben oder verschollen) | Yes |
| Germany Berlin | Extremely rare or restricted (Extrem selten) | No |
| Germany Berlin | Not assessed (Nicht einstufbar) | No |
| Germany Brandenburg | Not threatened (Nicht gefährdet) | No |
| Germany Hessen | Declining species (Vorwarnliste) | No |
| Germany Hessen | Restricted or extremely rare (Geographische Restriktion oder extrem selten) | No |
| Germany Nordrhein-Westfalen | Critically endangered but thanks to protective measures equal, less or no longer at risk (geringer oder nicht mehr gefährdet) | Yes |
| Germany Nordrhein-Westfalen | Data deficit (Daten unzureichend) | No |
| Germany Nordrhein-Westfalen | Data deficit(Daten unzureichend) | No |
| Germany Nordrhein-Westfalen | Declining (Vorwarnliste) | No |
| Germany Nordrhein-Westfalen | Declining but thanks to protective measures equal, less or no longer at risk (geringer oder nicht mehr gefährdet) | No |
| Germany Nordrhein-Westfalen | Endangered but thanks to protective measures equal, less or no longer at risk (geringer oder nicht mehr gefährdet) | Yes |
| Germany Nordrhein-Westfalen | Extremely rare or restricted (durch extreme Seltenheit (potentiell) gefährdet) | No |
| Germany Nordrhein-Westfalen | Extremely rare or restricted but thanks to protective measures equal, less or no longer at risk (geringer oder nicht mehr gefährdet) | No |
| Germany Nordrhein-Westfalen | Thanks to protective measures now not threatened (geringer oder nicht mehr gefährdet) | No |
| Germany Nordrhein-Westfalen | Threatened but thanks to protective measures equal, less or no longer at risk (geringer oder nicht mehr gefährdet) | Yes |
| Germany Nordrhein-Westfalen | Threatened status assumed (Gefährdung unbekannten Ausmaßes) | Yes |
| Germany, Germany Baden-Wuerttemberg | Data deficient (Datenlage unklar) | No |
| Germany, Germany Baden-Wuerttemberg, Germany Bayern | Possibly endangered (Gefährdung anzunehmen) | Yes |
| Germany, Germany Baden-Wuerttemberg, Germany Bayern, Germany Berlin, Germany Brandenburg, Germany Hessen, Germany Nordrhein-Westfalen | Critically endangered (Stark gefährdet) | Yes |
| Germany, Germany Baden-Wuerttemberg, Germany Bayern, Germany Berlin, Germany Brandenburg, Germany Hessen, Germany Nordrhein-Westfalen | Endangered (Gefährdet) | Yes |
| Germany, Germany Baden-Wuerttemberg, Germany Bayern, Germany Berlin, Germany Brandenburg, Germany Hessen, Germany Nordrhein-Westfalen | Threatened with extinction (Vom Aussterben bedroht) | Yes |
| Germany, Germany Baden-Wuerttemberg, Germany Bayern, Germany Hessen, Germany Nordrhein-Westfalen | Exinct or missing (Ausgestorben oder verschollen) | Yes |
| Germany, Germany Baden-Wuerttemberg, Germany Brandenburg | Extremely rare (Extrem selten) | No |
| Honduras | Amenazadas/Threatened, Importancia para ecoturismo/Importance for ecotourism | Yes |
| Honduras | Apéndices II CITES/Appendice II CITES | No |
| Honduras | Apéndices II CITES/Appendice II CITES, Importancia para ecoturismo/Importance for ecotourism | No |
| Honduras | Apéndices II CITES/Appendice II CITES, Raro/Rare, Importancia para ecoturismo/Importance for ecotourism | No |
| Honduras | Declining | No |
| Honduras | En peligro/In danger, Importancia para ecoturismo/Importance for ecotourism | Yes |
| Honduras | Endémica/Endemic, Raro/Rare, Importancia científica/Scientific importance, Apéndices I, II, III CITES/Appendices I, II, III CITES | No |
| Honduras | II SPAW Convention for the Protection and Development of the Marine Environment of the Wider Caribbean, Raro/Rare | No |
| Honduras | Importancia comercial o de consumo/Consumer or commercial importance, Importancia para ecoturismo/Importance for ecotourism | No |
| Honduras | Importancia cultural/Cultural significance | No |
| Honduras | Importancia para ecoturismo/Importance for ecotourism | No |
| Honduras | Migratoria/Migratory, Importancia para ecoturismo/Importance for ecotourism | No |
| Honduras | No Data | No |
| Honduras | No Evaluation | No |
| Honduras | Raro/Rare | No |
| Honduras | Raro/Rare, Importancia comercial o de consumo/Consumer or commercial importance | No |
| Honduras | Raro/Rare, Importancia para ecoturismo/Importance for ecotourism | No |
| Honduras | Stable | No |
| Ieland | Lower Risk | No |
| Ieland | Regionally Extinct in the Wild | Yes |
| India | Critically Endangered; C2a(i) | Yes |
| India | Critically Endangered downgraded to Endangered; B1ab(iii)+2ab(iii) | Yes |
| India | Critically Endangered; B1ab(ii, iii)+2ab(ii, iii) | Yes |
| India | Critically Endangered; B1ab(iii);D | Yes |
| India | Endangered upgraded to Critically Endangered; B1ab(i,ii,iii,iv,v); C1+2a | Yes |
| India | Endangered upgraded to Critically Endangered; B2ab(i,ii,iii,iv,v); C1+2a(i) | Yes |
| India | Endangered. Downgraded to Vulnerable; B1ab(iii)+2ab(iii) | Yes |
| India | Endangered; B1ab(iii), B2ab(iii) | Yes |
| India | Vulnerable . Downgraded to Near Threatened; B1ab(iii)+2ab(iii) | Yes |
| India | Vulnerable downgraded to Near Threatened; B1ab(ii, iii)+2ab(ii, iii) | Yes |
| India | Vulnerable downgraded to Near Threatened; B1ab(iii) + 2ab(iii) | Yes |
| India | Vulnerable. Downgraded to Near Threatened; B2ab(ii,iii) | Yes |
| India | Vulnerable. Downgraded to Near Threatened; B2ab(iii) | Yes |
| India | Vulnerable; A2c+3c+4c; B1ab(ii,iii) | Yes |
| India | Vulnerable; B2ab(ii, iii) | Yes |
| India Bihar, India Goa, India Himachal Pradesh, India Karnataka, India Kerala, India Madhya Pradesh, India Meghalaya, India Mizoram, India Orissa, India Punjab, India Tamil Nadu, India Tripura, India Uttarakhand, India West Bengal | On the verge of extinction | Yes |
| India, Israel, Pakistan, South Asia, Sri Lanka, Sweden, Switzerland | Endangered; D | Yes |
| India, Nepal | Critically Endangered; B1ab(iii,v)+2ab(iii,v); D | Yes |
| India, Nepal | Endangered. Downgraded to Vulnerable; B2ab(iii) | Yes |
| India, Nepal, Pakistan | Critically Endangered; B1ab(iii)+2ab(iii) | Yes |
| India, Nepal, Pakistan, South Asia, Sri Lanka | Endangered; B1ab(iii)+2ab(iii) | Yes |
| India, Nepal, South Asia, Sri Lanka | Vulnerable; B1ab(iii)+2ab(iii) | Yes |
| India, Nepal, Sri Lanka | Endangered; B2ab(ii,iii) | Yes |
| India, Nepal, Venezuela | Endangered; B2ab(iii) | Yes |
| India, Nepal, Venezuela | Vulnerable; D2 | Yes |
| India, Pakistan | Endangered downgraded to Vulnerable; B1ab(ii, iii)+2ab(ii, iii) | Yes |
| India, Pakistan | Vulnerable downgraded to Near Threatened; B2ab (ii,iii) | Yes |
| India, Pakistan | Vulnerable. Downgraded to Near Threatened; B1ab(ii,iii)+2ab(ii,iii) | Yes |
| India, Pakistan, South Asia | Endangered downgraded to Vulnerable; B2ab(iii) | Yes |
| India, South Asia | Endangered; A2abcd+3bcd; C1+2a(i) | Yes |
| India, South Asia | Endangered; A2c+3c+4c | Yes |
| India, South Asia | Endangered; B1ab(i,ii,iii)+2ab(i,ii,iii); C2a(i) | Yes |
| India, South Asia | Endangered; B2ab(i,ii,iii,iv,v) | Yes |
| India, South Asia | Endangered; B2ab(ii, iii) | Yes |
| India, South Asia | Endangered; C1+2a(i) | Yes |
| India, South Asia | Vulnerable downgraded to Near Threatened; B2ab(ii, iii) | Yes |
| India, South Asia | Vulnerable; A3c+4c | Yes |
| India, South Asia | Vulnerable; B2ab(ii,iii) | Yes |
| India, South Asia | Vulnerable; B2ab(iii,iv) | Yes |
| India, South Asia, Venezuela | Vulnerable; B1ab(iii) | Yes |
| India, Sri Lanka | Endangered; B1ab(ii, iii)+2ab(ii, iii) | Yes |
| India, Sri Lanka | Endangered; B2ab(ii,iii,iv) | Yes |
| India, Sweden | Endangered; B2ab(i,ii,iii,iv,v); C2a(i); D | Yes |
| India, Venezuela | Critically Endangered; B1ab(iii) | Yes |
| India, Venezuela | Endangered; B1ab(ii,iii)+2ab(ii,iii) | Yes |
| Israel | Critically Endangered; A1a,BD | Yes |
| Israel | Critically Endangered; A1ac,B1+2abcde,C1+2a,D | Yes |
| Israel | Critically Endangered; A1ac,B1+2abcde+3acd,C2ab,D | Yes |
| Israel | Critically Endangered; A1ac,BC | Yes |
| Israel | Critically Endangered; A1ac,BD | Yes |
| Israel | Critically Endangered; A1ac,D | Yes |
| Israel | Critically Endangered; A1acd,B1+2abcde,C1+2a,D | Yes |
| Israel | Critically Endangered; A1acd,C1+2a,D | Yes |
| Israel | Critically Endangered; A1ace,BD | Yes |
| Israel | Critically Endangered; A1c | Yes |
| Israel | Critically Endangered; A1c2c,E | Yes |
| Israel | Critically Endangered; A2acd,B1+2abcde,C1+2a,D | Yes |
| Israel | Critically Endangered; A4a,C | Yes |
| Israel | Critically Endangered; B | Yes |
| Israel | Critically Endangered; B,D | Yes |
| Israel | Critically Endangered; B1 | Yes |
| Israel | Critically Endangered; B1+2abcde,C2a | Yes |
| Israel | Critically Endangered; B1+2abcde,C2a,D | Yes |
| Israel | Critically Endangered; B1+3abcd,C2ab | Yes |
| Israel | Critically Endangered; C | Yes |
| Israel | Critically Endangered; C1+2a | Yes |
| Israel | Endangered; A1acd,B1+2abcde,C1+2a,D | Yes |
| Israel | Endangered; A1acd,B1+2abcde,C2a,D | Yes |
| Israel | Endangered; A1acd,B2abcde+3abde,C1 | Yes |
| Israel | Endangered; A1acd,C1+2a,D | Yes |
| Israel | Endangered; A1acde,C2b | Yes |
| Israel | Endangered; A1c | Yes |
| Israel | Endangered; A4a,B,C | Yes |
| Israel | Endangered; A4a,C | Yes |
| Israel | Endangered; A4ac,B | Yes |
| Israel | Endangered; A4ac,B,C | Yes |
| Israel | Endangered; A4ce,BC | Yes |
| Israel | Endangered; B | Yes |
| Israel | Endangered; B,C | Yes |
| Israel | Endangered; B,D | Yes |
| Israel | Endangered; B1 | Yes |
| Israel | Endangered; B1+2abcde | Yes |
| Israel | Endangered; B2abcde+3ae,C1+2ab | Yes |
| Israel | Endangered; B2b,C | Yes |
| Israel | Endangered; B4,D | Yes |
| Israel | Endangered; BD | Yes |
| Israel | Regionally Extinct + reintroduced | Yes |
| Israel | Regionally Extinct as breeder | Yes |
| Israel | Vulnerable; A1a,C1+2b | Yes |
| Israel | Vulnerable; A1acd,B2abc+3abde,C1+2b,D1 | Yes |
| Israel | Vulnerable; A1acd,C1 | Yes |
| Israel | Vulnerable; A1acd,C1,D1 | Yes |
| Israel | Vulnerable; A1acd,C1+2a,D1 | Yes |
| Israel | Vulnerable; A1acd,C1+2a,D1+2 | Yes |
| Israel | Vulnerable; A1acd+B1+2abcde,C1a | Yes |
| Israel | Vulnerable; A1acde,C1 | Yes |
| Israel | Vulnerable; A1c | Yes |
| Israel | Vulnerable; A1c,B | Yes |
| Israel | Vulnerable; A1cB | Yes |
| Israel | Vulnerable; A1e | Yes |
| Israel | Vulnerable; A4c,B | Yes |
| Israel | Vulnerable; B | Yes |
| Israel | Vulnerable; B,C | Yes |
| Israel | Vulnerable; B,C2b,D2 | Yes |
| Israel | Vulnerable; B,D1 | Yes |
| Israel | Vulnerable; B,D1+2 | Yes |
| Israel | Vulnerable; B1,C2a,D1+2 | Yes |
| Israel | Vulnerable; B1+2abc | Yes |
| Israel | Vulnerable; B2a,C1+2a | Yes |
| Israel | Vulnerable; B2b,C2b | Yes |
| Israel | Vulnerable; BC2b | Yes |
| Israel | Vulnerable; C2b,D1+2 | Yes |
| Israel | Vulnerable; C2b,D2 | Yes |
| Israel | Vulnerable; D1+2 | Yes |
| Israel, Norway | Critically Endangered; C1 | Yes |
| Israel, Sweden | Critically Endangered; D | Yes |
| Israel, Venezuela | Critically Endangered; A2ace | Yes |
| Japan | (Regionally) Extinct in the Wild | Yes |
| Japan | Locally Threatened Population | Yes |
| Japan | Threatened Local Population | Yes |
| Japan, Mediterranean Basin, Sri Lanka | (Regionally) Extinct | Yes |
| Lithuania | Extinct or possibly extinct | Yes |
| Lithuania | Restored | No |
| Maldives | Critically Endangered; B1ab(iii), B2ab(iii) | Yes |
| Mongolia | Critically Endangered, A2 | Yes |
| Mongolia | Critically Endangered, D | Yes |
| Mongolia | Endangered A3c | Yes |
| Mongolia | Endangered, A2acde | Yes |
| Mongolia | Endangered, A2acde and A3 | Yes |
| Mongolia | Endangered, A2ad | Yes |
| Mongolia | Endangered, A2cd and A3d | Yes |
| Mongolia | Endangered, A3c | Yes |
| Mongolia | Endangered, A3d | Yes |
| Mongolia | Endangered, A4abd | Yes |
| Mongolia | Endangered, A4cde | Yes |
| Mongolia | Endangered, B1ab(iii) | Yes |
| Mongolia | Endangered, C1 | Yes |
| Mongolia | Vulnerable, A3c | Yes |
| Mongolia | Vulnerable, A3cd | Yes |
| Mongolia | Vulnerable, A3cde | Yes |
| Mongolia | Vulnerable, B1ab(iii) | Yes |
| Mongolia | Vulnerable, D1 | Yes |
| Mongolia | Vulnerable, D2 | Yes |
| Nepal | Critically Endangerd | Yes |
| Nepal | Critically Endangered; B1ab(ii,iii,iv)+2ab(ii,iii,iv) | Yes |
| Nepal | Critically Endangered; B2ab(iii) | Yes |
| Nepal | Endangered upgraded to Critically Endangered | Yes |
| Nepal | Endangered. Downgraded to Vulnerable; B1ab(ii,iii)+2ab(ii,iii) | Yes |
| Nepal | Least Concerned | No |
| Nepal | Vulnerable . Downgraded to Near Threatened; B1ab(ii,iii,v)+2ab(ii,iii,v) | Yes |
| Nepal, Venezuela | Endangered; B1ab(iii) | Yes |
| Netherlands | (BE - Dutch criteria), (CR - IUCN criteria) | Yes |
| Netherlands | (BE - Dutch criteria), (EN - IUCN criteria) | Yes |
| Netherlands | (BE - Dutch criteria), (LC - IUCN criteria) | No |
| Netherlands | (BE - Dutch criteria), (NT - IUCN criteria) | No |
| Netherlands | (BE - Dutch criteria), (VU - IUCN criteria) | Yes |
| Netherlands | (EB - Dutch criteria), (CR - IUCN criteria) | Yes |
| Netherlands | (EB - Dutch criteria), (EN - IUCN criteria) | Yes |
| Netherlands | (EB - Dutch criteria), (VU - IUCN criteria) | Yes |
| Netherlands | (EN - IUCN criteria) | Yes |
| Netherlands | (GE - Dutch criteria), (CR - IUCN criteria) | Yes |
| Netherlands | (GE - Dutch criteria), (EN - IUCN criteria) | Yes |
| Netherlands | (GE - Dutch criteria), (LC - IUCN criteria) | No |
| Netherlands | (GE - Dutch criteria), (NT - IUCN criteria) | No |
| Netherlands | (GE - Dutch criteria), (VU - IUCN criteria) | Yes |
| Netherlands | (KW - Dutch criteria), (CR - IUCN criteria) | Yes |
| Netherlands | (KW - Dutch criteria), (EN - IUCN criteria) | Yes |
| Netherlands | (KW - Dutch criteria), (LC - IUCN criteria) | No |
| Netherlands | (KW - Dutch criteria), (NT - IUCN criteria) | No |
| Netherlands | (KW - Dutch criteria), (VU - IUCN criteria) | Yes |
| Netherlands | (NT - IUCN criteria) | No |
| Netherlands | (TNB - Dutch criteria) | No |
| Netherlands | (TNB - Dutch criteria), (EN - IUCN criteria) | Yes |
| Netherlands | (TNB - Dutch criteria), (NT - IUCN criteria) | No |
| Netherlands | (TNB - Dutch criteria), (VU - IUCN criteria) | Yes |
| Netherlands | (VN - Dutch criteria), (RE - IUCN criteria) | Yes |
| Netherlands | (VNW - Dutch criteria), (REW - IUCN criteria) | Yes |
| Netherlands | (VU - IUCN criteria) | Yes |
| New Zealand | 1 Nationally critical | Yes |
| New Zealand | 1 Nationally critical; Status criteria: 1 | Yes |
| New Zealand | 1 Nationally critical; Status criteria: 1, 2a | Yes |
| New Zealand | 1 Nationally critical; Status criteria: 1, 2a,b | Yes |
| New Zealand | 1 Nationally critical; Trend criteria: 3 | Yes |
| New Zealand | 2 Nationally endangered | Yes |
| New Zealand | 2 Nationally endangered; Status criteria: A1,2b; Trend criteria: A1 | Yes |
| New Zealand | 2 Nationally endangered; Status criteria: A1; Trend criteria: A1 | Yes |
| New Zealand | 2 Nationally endangered; Status criteria: A1; Trend criteria: A2 | Yes |
| New Zealand | 2 Nationally endangered; Status criteria: A2b; Trend criteria: A1 | Yes |
| New Zealand | 2 Nationally endangered; Status criteria: B1, 2a; Trend criteria: B1 | Yes |
| New Zealand | 2 Nationally endangered; Status criteria: B1; Trend criteria: B1 | Yes |
| New Zealand | 2 Nationally endangered; Status criteria: B1; Trend criteria: Special case | Yes |
| New Zealand | 2 Nationally endangered; Status criteria: B2b; Trend criteria: B1 | Yes |
| New Zealand | 3 Nationally vulnerable; Status criteria: 1; Trend criteria: 1 | Yes |
| New Zealand | 3 Nationally vulnerable; Status criteria: 2b; Trend criteria: 2 | Yes |
| New Zealand | 3 Nationally vulnerable; Status criteria: A1; Trend criteria: A1 | Yes |
| New Zealand | 4 Serious decline; Status criteria: A1; Trend criteria: A1 | No |
| New Zealand | 4 Serious decline; Status criteria: B2b; Trend criteria: B1 | No |
| New Zealand | 5 Gradual decline | No |
| New Zealand | 5 Gradual decline; Status criteria: 1, 2 a; Trend criteria: 1 | No |
| New Zealand | 5 Gradual decline; Status criteria: 1; Trend criteria: 1 | No |
| New Zealand | 6 Sparse | No |
| New Zealand | 7 Range restricted | No |
| New Zealand | 7 Range restricted; Status criteria: 1 | No |
| New Zealand | 8 Data deficient | No |
| New Zealand | Coloniser | No |
| New Zealand | Migrant | No |
| New Zealand | Not threatened | No |
| New Zealand | Vagrant | No |
| Nicaragua | Casi Amenazada (CA)/Near Threatened (NT) | Yes |
| Nicaragua | En Peligro (EP)/Endangered (EN) | Yes |
| Nicaragua | Peligro Crítico (PC)/Critically Endangered (CR) | Yes |
| Nicaragua | Posiblemente Extinta (PE)/Possibly Extinct | Yes |
| Nicaragua | Preocupación Menor (PM)/Least Concern (LC) | No |
| Nicaragua | Vulnerable (VU) | Yes |
| Norway | Critically Endangered; A2ab | Yes |
| Norway | Critically Endangered; B1b(iii)c(v)+2b(iii)c(v); C1+2a(i,ii)b; D1 | Yes |
| Norway | Critically Endangered; C2a(i); D1 | Yes |
| Norway | Endangered; A2bc; C1 | Yes |
| Norway | Vulnerable; A2a; C1 | Yes |
| Norway | Vulnerable; A2b | Yes |
| Norway | Vulnerable; A3d | Yes |
| Norway | Vulnerable; A4b | Yes |
| Norway, South Asia | Vulnerable; A2a | Yes |
| Norway, Sweden | Vulnerable; A2bc | Yes |
| Norway, Sweden, Switzerland | Endangered; C1 | Yes |
| Norway, Switzerland, Venezuela | Vulnerable; A2c | Yes |
| Norway, Venezuela | Critically Endangered; C2a(ii) | Yes |
| Pakistan | Critically Endangered; B1ab(ii, iii) | Yes |
| Pakistan | Endangered downgraded to Vulnerable; B1ab (ii,iii)+2ab (ii,iii) | Yes |
| South Asia | Critically Endangered; B1ab(iii,v)+2ab(iii,v) | Yes |
| South Asia | Endangered; B1ab(iii) + 2ab(iii) | Yes |
| South Asia | Endangered; B1ab(iii)+2ab (iii) | Yes |
| South Asia | Endangered; B2ab(ii, iii, iv) | Yes |
| South Asia | Endangered; C1+2a(i); D | Yes |
| South Asia | Vulnerable downgraded to Near Threatened; B2ab(iii) | Yes |
| South Asia | Vulnerable downgraded to Near Threatened; B2ab(iii);D2 | Yes |
| South Asia | Vulnerable; A2c+3c+4c; B1ab(ii, iii) | Yes |
| South Asia | Vulnerable; A2c+3c+4c; D | Yes |
| South Asia | Vulnerable; B2ab(ii, iii, iv) | Yes |
| South Asia | Vulnerable; B2ab(iii); D2 | Yes |
| Sri Lanka | Critically Endangered, B1ab (iii) | Yes |
| Sri Lanka | Critically Endangered, B1ab(i,ii,iii) | Yes |
| Sri Lanka | Critically Endangered, B1ab(i,ii,iii)+2ab(i,ii,iii) | Yes |
| Sri Lanka | Critically Endangered, B1ab(i,ii,iii)c(i,ii) | Yes |
| Sri Lanka | Critically Endangered, B2ab(i,ii,iii) | Yes |
| Sri Lanka | Critically Endangered, B2ab(iii) | Yes |
| Sri Lanka | Endangered, B1ab(i,ii,iii)+2ab(i,ii,iii) | Yes |
| Sri Lanka | Endangered, B1ab(iii)+2ab(iii) | Yes |
| Sri Lanka | Endangered, B2ab(i,ii,iii) | Yes |
| Sri Lanka | Endangered, B2ab(iii) | Yes |
| Sri Lanka | Endangered, B2b(i,ii,iii)c(iii) | Yes |
| Sri Lanka | Endangered; A2cd+4cd | Yes |
| Sri Lanka | Endangered; B1ab(ii, iii) | Yes |
| Sri Lanka | Endangered; B2ab(ii,iii,v) | Yes |
| Sri Lanka | Vulnerable, B1ab(i,ii,iii)+2ab(i,ii,iii) | Yes |
| Sri Lanka | Vulnerable, B1ab(iii)+2ab(iii) | Yes |
| Sri Lanka | Vulnerable, B1b(iii)c(iii)+2b(iii)c(iii) | Yes |
| Sri Lanka | Vulnerable, B1b(iii)c(iii,iv)+2b(iii)c(iii,iv) | Yes |
| Sri Lanka | Vulnerable, B2ab(i,ii,iii) | Yes |
| Sri Lanka | Vulnerable, B2b(i,ii,iii)c(i, iii, iv) | Yes |
| Sri Lanka | Vulnerable, B2b(i,ii,iii)c(iii) | Yes |
| Sri Lanka | Vulnerable; A3c+4c;B1ab(ii, iii) | Yes |
| Sri Lanka | Vulnerable; B1ab(ii, iii)+2ab(ii, iii) | Yes |
| Sri Lanka | Vulnerable; B1ab(ii,iii)+2ab(ii,iii) | Yes |
| Sweden | Critically Endangered; A3c; B2ab(ii,iii,iv,v); C1 | Yes |
| Sweden | Critically Endangered; B2ab(v); D | Yes |
| Sweden | Critically Endangered; C1+2a(i,ii); D | Yes |
| Sweden | Endangered; A2bc | Yes |
| Sweden | Endangered; A2bce+4bce | Yes |
| Sweden | Endangered; A2bde | Yes |
| Sweden | Endangered; C2a (i) | Yes |
| Sweden | Endangered; C2a(i); D | Yes |
| Sweden | Endangered; C2a(i,ii); D | Yes |
| Sweden | Vulnerable; A2b; C1 | Yes |
| Sweden | Vulnerable; A2bc; C1 | Yes |
| Sweden | Vulnerable; A2bc; C1+2a(i) | Yes |
| Sweden | Vulnerable; A2bc+3bc+4bc | Yes |
| Sweden | Vulnerable; A2bc+3bc+4bc; C1 | Yes |
| Sweden | Vulnerable; A2bcd+3c+4bc | Yes |
| Sweden | Vulnerable; A2bcde | Yes |
| Sweden | Vulnerable; A2bcde; C1 | Yes |
| Sweden | Vulnerable; A2bce | Yes |
| Sweden | Vulnerable; A2bce; C1 | Yes |
| Sweden | Vulnerable; A2bde+3bde+4bde; C1 | Yes |
| Sweden | Vulnerable; A2be+3be+4be | Yes |
| Sweden | Vulnerable; B1ab(i,ii,iii,v)+2ab(i,ii,iii,v); C1 | Yes |
| Sweden | Vulnerable; B1ab(v)+2ab(v); C1+2a(i); D1 | Yes |
| Sweden | Vulnerable; C1; D1 | Yes |
| Sweden | Vulnerable; C1+2a(i) | Yes |
| Sweden, Switzerland | Vulnerable; D | Yes |
| Switzerland | Critically Endangered; B1,2ce C2a D | Yes |
| Switzerland | Critically Endangered; B1,2ce C2b D | Yes |
| Switzerland | Critically Endangered; B1,2e C2a D | Yes |
| Switzerland | Critically Endangered; B1,2e C2b D | Yes |
| Switzerland | Critically Endangered; C1,2a D | Yes |
| Switzerland | Critically Endangered; C2a D | Yes |
| Switzerland | Critically Endangered; C2b D | Yes |
| Switzerland | Endangered; A2c | Yes |
| Switzerland | Endangered; A2c, B2a, B2b(ii, iv) | Yes |
| Switzerland | Endangered; B2a, B2b(ii, iv) | Yes |
| Switzerland | Endangered; C2a D | Yes |
| Switzerland | Endangered; C2a D1 | Yes |
| Switzerland | Near Threatened; A1a | No |
| Switzerland | Near Threatened; A1c | No |
| Switzerland | Near Threatened; C1 | No |
| Switzerland | Near Threatened; cd | No |
| Switzerland | Near Threatened; D1, cd | No |
| Switzerland | Vulnerable; A2c, B2a, B2b(ii, iv) | Yes |
| Switzerland | Vulnerable; A3c,e | Yes |
| Switzerland | Vulnerable; C1 D1 | Yes |
| Switzerland | Vulnerable; C2a | Yes |
| Switzerland | Vulnerable; C2a D1 | Yes |
| Switzerland | Vulnerable; C2a(ii), D2 | Yes |
| United Kingdom | Amber | No |
| United Kingdom | Bird Population Status - amber | No |
| United Kingdom | Bird Population Status - red | Yes |
| United Kingdom | Bird Population Status: Amber (see Notes for further detail) | No |
| United Kingdom | Bird Population Status: Red (see Notes for further detail) | Yes |
| United Kingdom | Green | No |
| United Kingdom | Red | Yes |
| Uruguay | Endandered | Yes |
| Venezuela | Critically Endangered (En Peligro Crítico) | Yes |
| Venezuela | Critically Endangered; A2a; B2ab(iv) | Yes |
| Venezuela | Critically Endangered; A2ac; B1ab(iii,iv) | Yes |
| Venezuela | Critically Endangered; A2ace; B1ab(iii,iv)+2ab(iii,iv) | Yes |
| Venezuela | Critically Endangered; A2ace; B2ab(iii,iv) | Yes |
| Venezuela | Critically Endangered; A2ace; B2ab(iv) | Yes |
| Venezuela | Critically Endangered; A2cd | Yes |
| Venezuela | Critically Endangered; A3ce | Yes |
| Venezuela | Critically Endangered; B1ab(iv)+2ab(iv) | Yes |
| Venezuela | Endangered (En Peligro) | Yes |
| Venezuela | Endangered; A1abd | Yes |
| Venezuela | Endangered; A2cd | Yes |
| Venezuela | Endangered; A2cd; B1ab(i,iii) | Yes |
| Venezuela | Endangered; A3c | Yes |
| Venezuela | Endangered; A3cd | Yes |
| Venezuela | Endangered; B1ab(i,ii,iii); C2a(ii) | Yes |
| Venezuela | Endangered; B1ab(i,ii,iii,iv) | Yes |
| Venezuela | Endangered; B1ab(i,ii,iii,iv); C2a(i) | Yes |
| Venezuela | Endangered; B1ab(i,iii) | Yes |
| Venezuela | ER | Yes |
| Venezuela | Extinct (Extinto) | Yes |
| Venezuela | Regionally Extinct (Extinto a Nivel Regional) | Yes |
| Venezuela | Vulnerable; A1ad | Yes |
| Venezuela | Vulnerable; A1bd | Yes |
| Venezuela | Vulnerable; A1cd+2c; C1 | Yes |
| Venezuela | Vulnerable; A2acde+3de | Yes |
| Venezuela | Vulnerable; A2cd | Yes |
| Venezuela | Vulnerable; A2cd; B1,2ab(i,ii,iii,iv,v) | Yes |
| Venezuela | Vulnerable; A2d | Yes |
| Venezuela | Vulnerable; B1+2ab(iii) | Yes |
| Venezuela | Vulnerable; B1ab(i,ii,iii) | Yes |
| Venezuela | Vulnerable; B1ab(i,ii,iii); C2a(ii) | Yes |
| Venezuela | Vulnerable; B1ab(i,ii,iii,iv); C1+2a(ii) | Yes |
| Venezuela | Vulnerable; B1ab(i,iii) | Yes |
| Venezuela | Vulnerable; B1ab(i,iii); C2a(ii) | Yes |
| Venezuela | Vulnerable; B1ab(iii); D2 | Yes |
| Venezuela | Vulnerable; C2a(ii) | Yes |
| Venezuela | Vulnerable; C2a(ii); D1 | Yes |

**Fig. S1.1.** Schematic model of the various HANPP-related metrics. The human appropriation of net primary production (HANPP) is an indicator of the effects of human-induced changes in land use and the availability of biomass flows. HANPP is calculated as the sum of (1) the change in NPP due to human-induced land conversion and land use change (HANPP_luc_) and (2) the amount of NPP harvested or otherwise consumed by people (HANPP_harv_). Viewed differently, HANPP is the difference between the net primary productivity of the natural vegetation (NPP_pot_), i.e. before human land use, and current NPP (NPP_eco_), i.e. after land conversion and harvest. Due to irrigation and/or fertilization, NPP_eco_ levels can surmount those of NPP_pot_. (Source: adapted from Haberl et al. 2014).


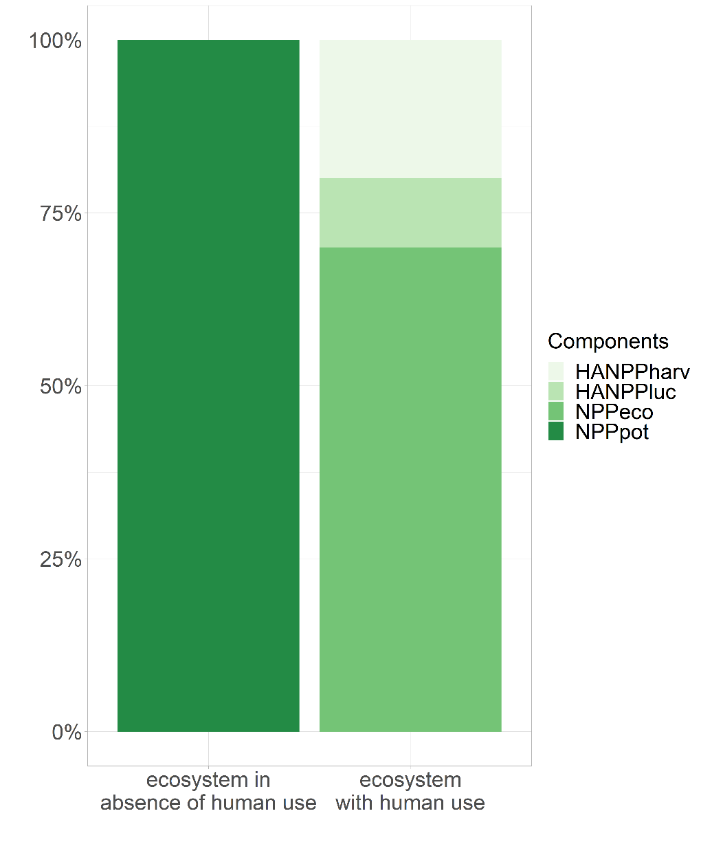


**Fig. S1.2.** Wilderness areas (in pale grey) and zoogeographic realms of the world. Realm 4 (Madagascar) was treated as no data zone as it contains no wilderness areas which did not allow for inferences about SR loss there (Data source wilderness areas: Venter et al. 2016a, 2016b; Potapov et al. 2017; Potapov et al. 2008, see Method section of main text for more detailed information; data source zoogeographic realms: Holt et al. 2013). Projection: Eckert IV.


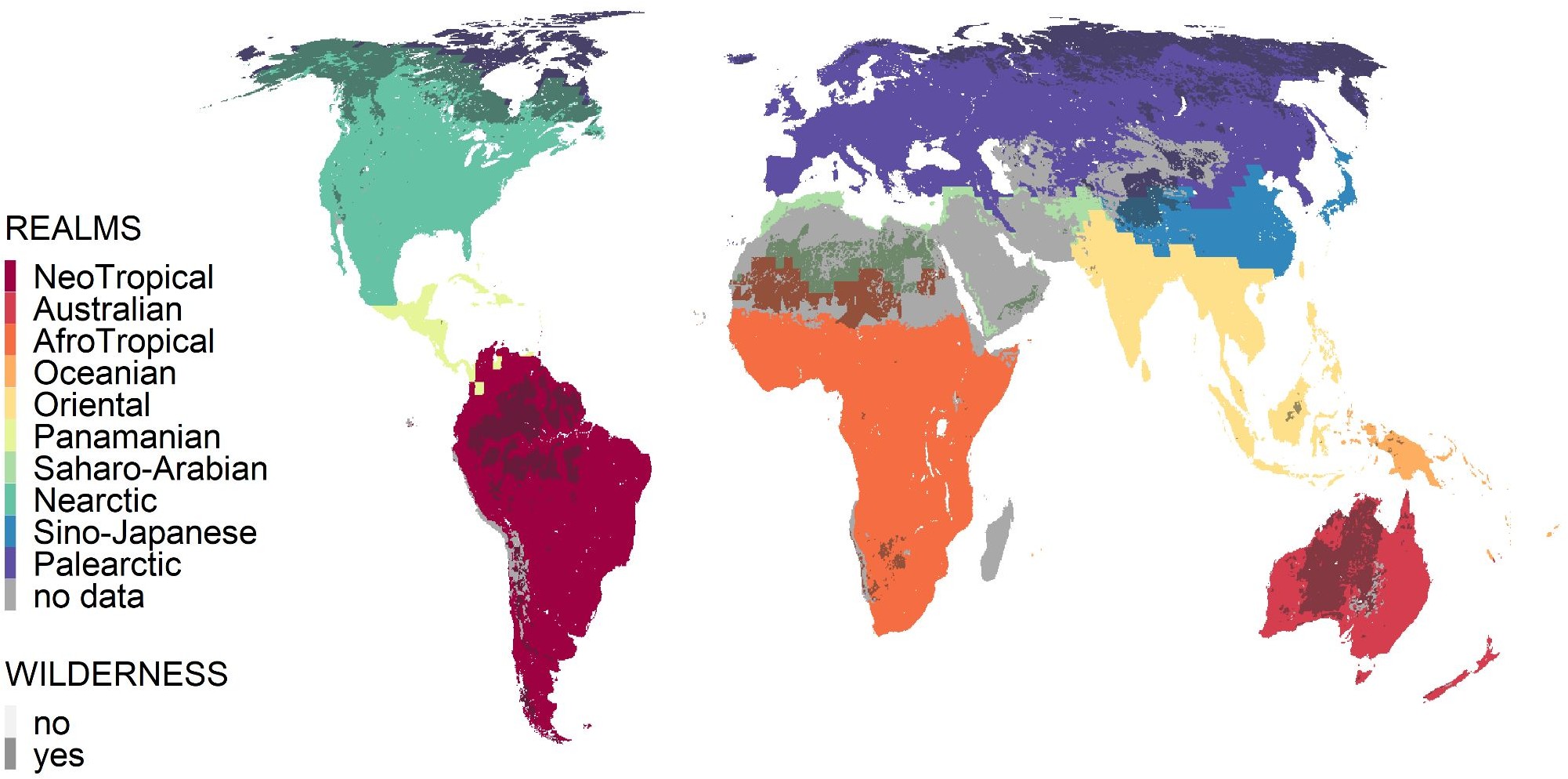


ForPeer

Review

**Fig. S1.3.** From top to bottom: maps of the amount of NPP_pot_ (NPP of the natural vegetation, i.e. vegetation that would prevail in a hypothetic case without land use, under current climate conditions), NPP_eco_ (NPP left after land conversion and harvest for human use) and the difference between both, i.e. HANPP (human appropriation of net primary production) in tons of carbon per year. Projection: Eckert IV.


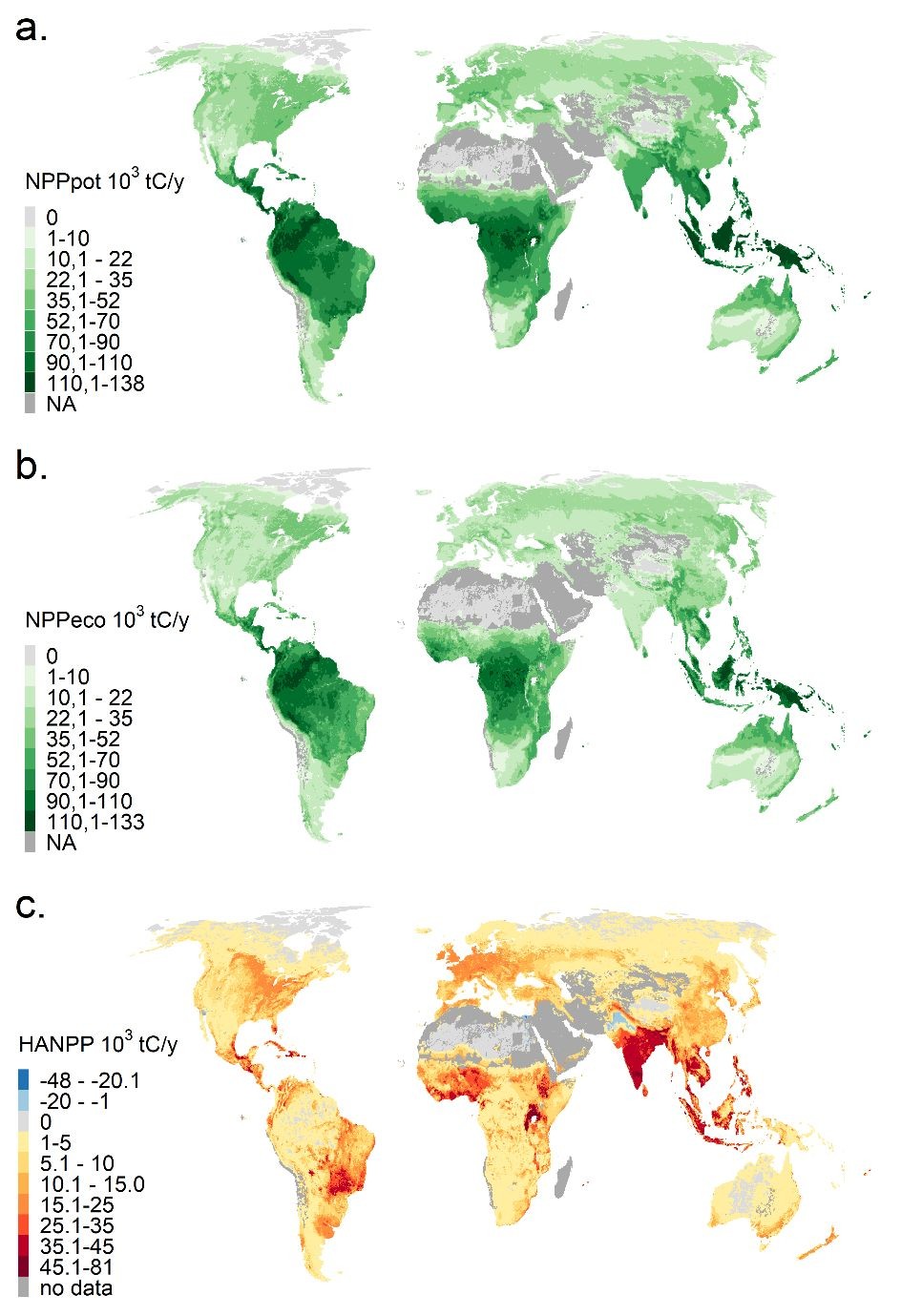


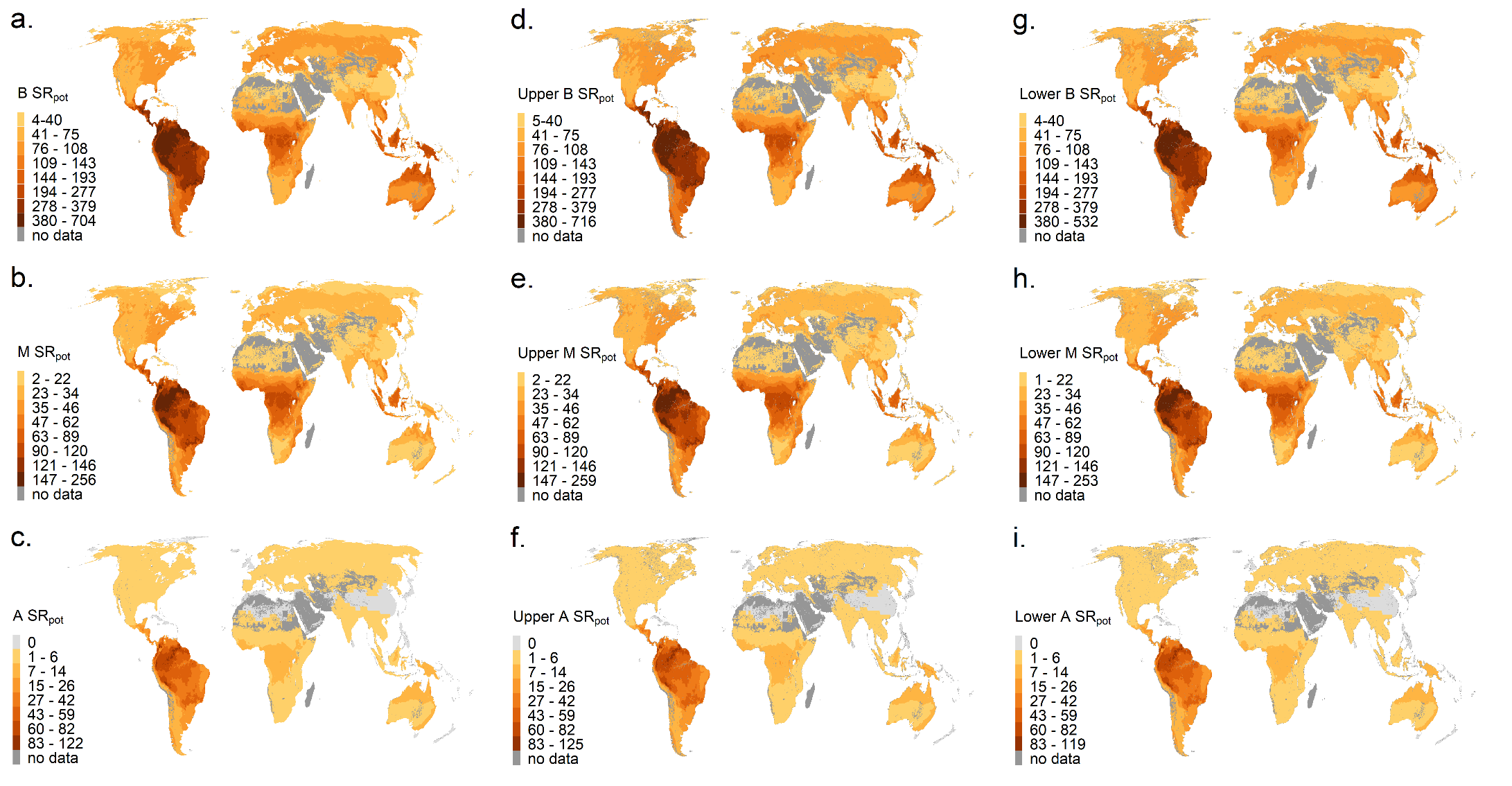
**Fig. S1.4.** Maps showing the pristine SR as projected by SER models (SR_pot_, i.e. richness under NPP_pot_) for birds, mammals and amphibians as predicted by our SER-models. Column 1 (i.e. figures a–c): projections based on the estimated regression coefficients; column 2 (i.e. figures d–f): projections based on the estimated regression coefficients plus their standard errors; column 3 (i.e. figures g–i): projections based on the estimated regression coefficients minus their standard errors.

**Fig. S1.5.** Scatterplot showing the relationship between the pristine SR as projected by SER models (SR_pot_) and NPP_pot_ across (1) 20,000 randomly selected 20x20 km cells for birds (a), mammals (b) and amphibians (c) (grey dots) and (2) the sample of 3,575 wilderness areas cells used for calibrating the model (green dots). The orange line represents the partial effect of NPP_pot_ in the SER models, averaged across all biogeographical realms, i.e. the estimated marginal means (EMMs), computed by the emmeans-package (Lenth 2022). The mainland-island co-variable is kept constant, i.e. at level *mainland*, which represents the vast majority of data points in the overall data set.


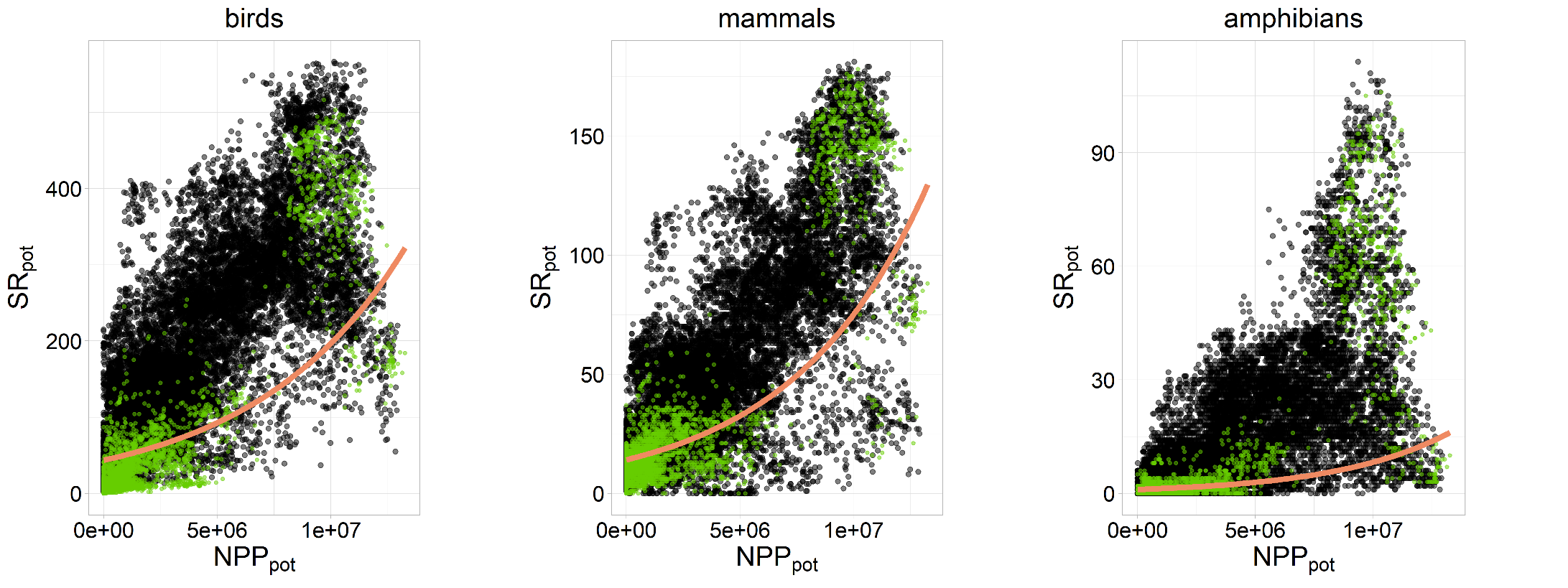


**Appendix 1 – Data Sources**

BirdLife International and Handbook of the Birds of the World (2018): Bird species distribution maps of the world. Available online at http://datazone.birdlife.org/species/requestdis.

Bjelle, Eivind Lekve; Többen, Johannes; Stadler, Konstantin; Kastner, Thomas; Theurl, Michaela C.; Erb, Karl-Heinz et al. (2020): Adding country resolution to EXIOBASE: impacts on land use embodied in trade. In Economic Structures 9 (1), p. 14. DOI: 10.1186/s40008-020-0182-y.

ESA (2017): Land Cover CCI Product User Guide Version 2. Tech. Rep. Available online at maps.elie.ucl.ac.be/CCI/viewer/download/ESACCI-LC-Ph2-PUGv2_2.0.pdf.

Food and Agriculture Organization of the United Nations (2020): FOASTAT. Statistical Databases. Rome, Italy. Available online at http://faostat.fao.org.

Haberl, Helmut; Erb, K. Heinz; Krausmann, Fridolin; Gaube, Veronika; Bondeau, Alberte; Plutzar, Christoph et al. (2007): Quantifying and mapping the human appropriation of net primary production in earth's terrestrial ecosystems. In Proceedings of the National Academy of United States of America 104 (31), pp. 12942–12947. DOI: 10.1073/pnas.0704243104.

Haberl, Helmut; Erb, Karl-Heinz; Krausmann, Fridolin (2014): Human Appropriation of Net Primary Production: Patterns, Trends, and Planetary Boundaries. In Annual Review of Environment and Resources 39 (1), pp. 363–391. DOI: 10.1146/annurev-environ-121912-094620.

Holt, Ben G.; Lessard, Jean-Philippe; Borregaard, Michael K.; Fritz, Susanne A.; Araújo, Miguel B.; Dimitrov, Dimitar et al. (2013): An update of Wallace's zoogeographic regions of the world. In Science (New York, N.Y.) 339 (6115), pp. 74–78. DOI: 10.1126/science.1228282.

IUCN (2020): The IUCN Red List of Threatened Species. Version 2020-2. Available online at http://www.iucnredlist.org.

Kastner, Thomas; Matej, Sarah; Forrest, Matthew; Gingrich, Simone; Haberl, Helmut; Hickler, Thomas et al. (2022): Land use intensification increasingly drives the spatiotemporal patterns of the global human appropriation of net primary production in the last century. In Global change biology 28 (1), pp. 307–322. DOI: 10.1111/gcb.15932.

Klein Goldewijk, Kees; Beusen, Arthur; Doelman, Jonathan; Stehfest, Elke (2017): Anthropogenic land use estimates for the Holocene – HYDE 3.2. In Earth Syst. Sci. Data 9 (2), pp. 927–953. DOI: 10.5194/essd-9-927-2017.

Krausmann, Fridolin; Erb, Karl-Heinz; Gingrich, Simone; Haberl, Helmut; Bondeau, Alberte; Gaube, Veronika et al. (2013): Global human appropriation of net primary production doubled in the 20th century. In Proceedings of the National Academy of Sciences of the United States of America 110 (25), pp. 10324–10329. DOI: 10.1073/pnas.1211349110.

Lenth, Russel V. (2022): emmeans: Estimated Marginal Means, aka Least-Squares Means. Version 1.7.2. Available online at https://CRAN.R-project.org/package=emmeans.

Nishina, Kazuya; Ito, Akihiko; Hanasaki, Naota; Hayashi, Seiji (2017): Reconstruction of spatially detailed global map of NH4+ and NO3−application in synthetic nitrogen fertilizer. In Earth Syst. Sci. Data 9 (1), pp. 149–162. DOI: 10.5194/essd-9-149-2017.

Oerke, E. C.; Dehne, H. W.; Schönbeck, F.; Weber, A. (1994): Crop Production and Crop Protection. Estimated Losses in Major Food and Cash Crops. In E. C. Oerke (Ed.): Crop Production and Crop Protection. Estimated Losses in Major Food and Cash Crops. Amsterdam: Elsevier.

Olson, D. M.; Dinerstein, E.; Wikramanayake, E. D.; Burgess, N. D.; Powell, G. V. N.; Underwood, E. C. et al. (2001): Terrestrial ecoregions of the world. a new map of life on Earth. In Bioscience 51 (11), pp. 933–938. Available online at URL: http://worldwildlife.org/publications/terrestrial-ecoregions-of-the-world.

Potapov, Peter; Hansen, Matthew C.; Laestadius, Lars; Turubanova, Svetlana; Yaroshenko, Alexey; Thies, Christoph et al. (2017): The last frontiers of wilderness: Tracking loss of intact forest landscapes from 2000 to 2013. In Sci. Adv. 3 (1), e1600821. DOI: 10.1126/sciadv.1600821.

Potapov, Peter; Yaroshenko, Alexey; Turubanova, Svetlana; Dubinin, Maxim; Laestadius, Lars; Thies, Christoph et al. (2008): Mapping the Worlds Intact Forest Landscapes by Remote Sensing. In Ecol. Soc. 13. DOI: 10.5751/ES-02670-130251.

Saugier, Bernard; Roy, Jacques; Mooney, Harold (2001): Estimations of global terrestrial productivity: Converging toward a single number? In: Terrestrial Global Productivity, pp. 543–557.

Semenchuk, Philipp; Plutzar, Christoph; Kastner, Thomas; Matej, Sarah; Bidoglio, Giorgio; Erb, Karl-Heinz et al. (in revision): Relative effects of land conversion and land-use intensity on terrestrial vertebrate diversity.

Venter, Oscar; Sanderson, Eric W.; Magrach, Ainhoa; Allan, James R.; Beher, Jutta; Jones, Kendall R. et al. (2016a): Global terrestrial Human Footprint maps for 1993 and 2009. In Sci Data 3 (1), p. 160067. DOI: 10.1038/sdata.2016.67.

Venter, Oscar; Sanderson, Eric W.; Magrach, Ainhoa; Allan, James R.; Beher, Jutta; Jones, Kendall R. et al. (2016b): Sixteen years of change in the global terrestrial human footprint and implications for biodiversity conservation. In Nat Commun 7 (1), p. 12558. DOI:10.1038/ncomms12558.

Wirsenius, Stefan (2000): Human use of land and organic materials: Modeling the turnover of biomass in the global food system. Doctoral Thesis. Chalmers University of Technology; Gäteborg University, Göteborg, Sweden. Department of Physical Resource Theory.

Zika, Michael; Erb, Karl-Heinz (2009): The global loss of net primary production resulting from human-induced soil degradation in drylands. In: Ecological Economics 69 (2), pp. 310–318. DOI: 10.1016/j.ecolecon.2009.06.014.
